# Perivascular tenascin C triggers sequential activation of macrophages and endothelial cells to generate a pro-metastatic vascular niche in the lungs

**DOI:** 10.1038/s43018-022-00353-6

**Published:** 2022-04-25

**Authors:** Tsunaki Hongu, Maren Pein, Jacob Insua-Rodríguez, Ewgenija Gutjahr, Greta Mattavelli, Jasmin Meier, Kristin Decker, Arnaud Descot, Matthias Bozza, Richard Harbottle, Andreas Trumpp, Hans-Peter Sinn, Angela Riedel, Thordur Oskarsson

**Affiliations:** 1 Heidelberg Institute for Stem Cell Technology and Experimental Medicine (HI-STEM gGmbH), Heidelberg, Germany; 2 Division of Stem Cells and Cancer, German Cancer Research Center, Heidelberg, Germany; 3 Faculty of Biosciences, University of Heidelberg, Heidelberg, Germany; 4 Institute of Pathology, University of Heidelberg, Heidelberg, Germany; 5 Mildred Scheel Early Career Center, University Hospital of Würzburg, Würzburg, Germany; 6 DNA Vector Laboratory, German Cancer Research Center, Heidelberg, Germany; 7 DKFZ-ZMBH Alliance, Heidelberg, Germany; 8 German Cancer Consortium, Heidelberg, Germany; 9 Present Address: Department of Molecular Oncology and Cancer Biology and Evolution Program, H. Lee Moffitt Cancer Center and Research Institute, Tampa, FL USA

**Keywords:** Breast cancer, Cancer microenvironment, Metastasis, Cancer

## Abstract

Disseminated cancer cells frequently lodge near vasculature in secondary organs. However, our understanding of the cellular crosstalk invoked at perivascular sites is still rudimentary. Here, we identify intercellular machinery governing formation of a pro-metastatic vascular niche during breast cancer colonization in the lung. We show that specific secreted factors, induced in metastasis-associated endothelial cells (ECs), promote metastasis in mice by enhancing stem cell properties and the viability of cancer cells. Perivascular macrophages, activated via tenascin C (TNC) stimulation of Toll-like receptor 4 (TLR4), were shown to be crucial in niche activation by secreting nitric oxide (NO) and tumor necrosis factor (TNF) to induce EC-mediated production of niche components. Notably, this mechanism was independent of vascular endothelial growth factor (VEGF), a key regulator of EC behavior and angiogenesis. However, targeting both macrophage-mediated vascular niche activation and VEGF-regulated angiogenesis resulted in added potency to curb lung metastasis in mice. Together, our findings provide mechanistic insights into the formation of vascular niches in metastasis.

## Abstract (web summary)

Hongu et al. find that perivascular macrophages stimulate activation of the pro-metastatic vascular niche via tenascin C stimulation of TLR4 and show that combined TLR4 and VEGF inhibition prevents TNC-mediated metastatic vascular activity.

## Main

When cancers progress to metastasis, interactions between disseminated cancer cells and nontransformed cells of the microenvironment play an important role^1^. Crosstalk between disseminated cancer cells and stromal cells of secondary organs can result in the generation of metastatic niches that promote malignant growth^2,3^. Endothelial cells are frequently prominent within the stroma of both primary tumors and metastases; they form the inner cell layer of vasculature, such as blood vessels that are crucial for cancer growth by delivering nutrients and other essentials to tumors^4^. Vascular endothelial growth factor is a central promoter of angiogenesis, the formation of new blood vessels from pre-existing ones, by induction of endothelial cell proliferation, migration and survival^5^. This can lead to increased vascular permeability and sprouting by activating tip cells of established vessels^4^. Recent findings indicate that blood vessels can have substantial impact on metastatic progression that extends beyond nutrient delivery^6^. Studies have shown that disseminated cancer cells associate with vasculature at metastatic sites, and suggest that enhanced adhesion and crosstalk with ECs regulate phenotype and function of cancer cells in metastasis^7–9^.

In this study, we analyzed interactions between breast cancer cells and ECs in the lung during metastatic progression. We identified components of a pro-metastatic vascular niche that are independent of VEGF signaling and support stem cell properties and survival of disseminated cancer cells. Exploring the regulatory mechanisms of vascular niche formation, we found that it was not directly induced by cancer cells but required metastasis-associated macrophages as intermediates. Perivascular macrophages, activated by the extracellular matrix protein TNC via TLR4, promote the formation of a vascular niche by secreting NO and TNF to induce expression of niche components in ECs. The results reveal a critical crosstalk within vascular niches, and underscore the importance of extracellular matrix proteins as regulators of the microenvironment in metastasis.

## Results

### Molecular reprogramming of ECs in lungs harboring metastases

**Fig. 1:**
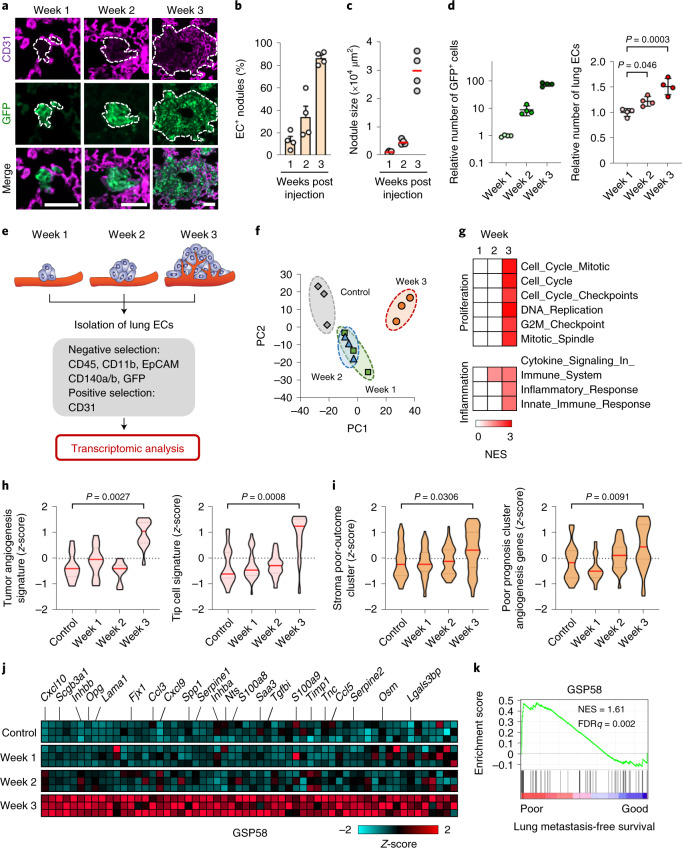
Transcriptomic analysis identifies characteristic changes in reactive ECs during metastatic colonization of the lung. **a**, Immunofluorescence images showing association of lung ECs (CD31) with metastatic breast cancer cells (GFP) in mouse lung at indicated time points post intravenous injection of MDA231-LM2 breast cancer cells. Scale bars, 50 μm. Dashed lines indicate margins of metastatic foci. **b**, Quantification of metastatic nodules from **a** with intranodular ECs; *n* = 72 nodules (week 1), *n* = 76 nodules (week 2) and *n* = 83 nodules (week 3) from four mice were analyzed for each time point. Data are presented as means ± s.e.m. **c**, Size of MDA231-LM2-derived metastatic nodules in lung at weeks 1–3. A minimum of 16 nodules were analyzed for each lung; *n* = 4 mice per group. **d**, MDA231-LM2 cancer cells (left) and ECs (right) in lung at indicated time points. Data show means ± s.d; *n* = 4 mice per time point. *P* values were determined by one-way ANOVA with Dunnett’s multiple comparison test. **e**, Experimental setup for EC isolation from mouse lung at different stages of MDA231-LM2-derived metastasis, followed by transcriptomic analysis. **f**, PC analysis of gene expression profiles from ECs isolated from healthy lung (control) or lung with different stages of metastasis (as in **e**). **g**, GSEA of isolated ECs using proliferation- or inflammation-related signatures. Signatures with nominal *P* < 0.05 and FDR < 0.25 were considered significant. **h**,**i**, Violin plots showing *z*-score analysis of tumor angiogenesis and tip cell signatures^11,12^ (**h**) or patient poor-outcome gene clusters^13,14^ (**i**), calculated from transcriptomic profiles of ECs isolated from metastatic lungs at indicated time points. *P* values were determined using averaged *z*-scores of each signature by unpaired two-tailed *t*-test; *n* = 3 biological replicates per group. **j**, Heatmap showing expression of 58 genes of secreted proteins (GSP58) upregulated in lung ECs at week 3 post cancer cell injection. Cutoff log_2_(fold change (FC)) > 0.75, *P* < 0.05, FDR*q* < 0.25. **k**, GSEA graph showing enrichment of GSP58 in samples of human lung metastases of breast cancer, ranked according to lung metastasis-free survival. NES, normalized enrichment score. FDR was determined from *P* values calculated by random permutation test. Source data

**Extended Data Fig. 1:**
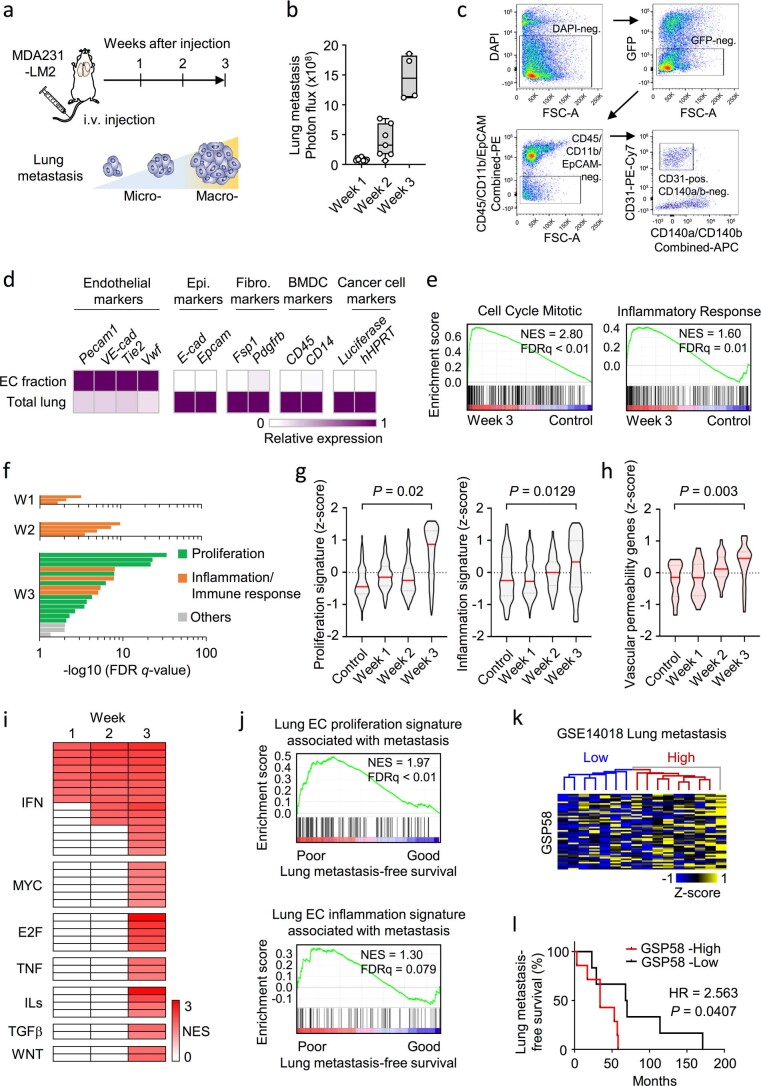
Isolation and transcriptional analysis of metastasis-associated ECs. **a**,**b** MDA231-LM2 cells were injected intravenously into NSG mice and lung metastasis analyzed at week 1, week 2 and week 3 post injection. Lung colonization determined by bioluminescence in mice at indicated time points; *n*= 11 (week 1), *n*= 7 (week 2), *n*= 4 (week 3). Boxes show median with upper and lower quartiles and whiskers indicate maximum and minimum values. **c**, FACS plots showing isolation of ECs from lungs with metastasis. **d**, Heatmap displaying expression of cell type-specific markers in total lung cells or sorted EC fractions from lungs containing MDA231-LM2 metastases. Endothelial cell-, epithelial cell-, fibroblast-, bone marrow-derived cell- (BMDC), and human cancer cell markers were analyzed; *n*= 3 experiments. **e**, GSEA addressing proliferation (“Cell cycle mitotic” in REACTOME, left) and inflammation (“Inflammatory response” in Hallmark of MSigDB, right) in ECs isolated from mouse lungs harboring metastases. FDR was determined from *P* values calculated by random permutation test. **f**, Gene ontology (GO) analysis of upregulated genes (FDR< 0.05, log2FC> 1) within metastatic EC transcriptome at indicated time points. Biological processes with FDR< 0.05 are shown. Specific GO terms in graph are listed in Supplementary Table 1. **g**,**h**, Violin plot analysis of z-scores from gene signatures for proliferation (Cell cycle mitotic, REACTOME), inflammation (Inflammatory response, Hallmark in MSigDB) and vascular permeability genes (VEGFR2-mediated vascular permeability, REACTOME). Z-scores were calculated from gene expression profiles of lung ECs isolated from metastatic lungs at indicated times. *P* values were determined by unpaired two-tailed t-test, using z-score averages from 3 biological replicates. **i**, Heatmap showing GSEA of signaling pathways (C2 collection of MSigDB) enriched in ECs at indicated time points. Signatures with nominal *P*< 0.05 and FDR< 0.25 were included. NES, normalized enrichment score. **j**, GSEA showing enrichment of EC proliferation signature (top, Supplementary Table 3) and EC inflammation signature (bottom, Supplementary Table 4) in human metastases samples with poor lung metastasis-free survival. **k**, Hierarchical clustering of lung metastasis samples from breast cancer patients (GSE14018) according to expression of GSP58; *n*=16 patients. **l**, Kaplan-Meier analysis of lung metastasis-free survival in breast cancer patients stratified according to expression of GSP58 in metastatic nodules. GSP58-High, *n*= 7; GSP58-Low, *n*= 6. *P* value was determined by log-rank test. HR, hazard ratio. Source data

To investigate the molecular changes in ECs during metastatic colonization, we isolated ECs from lungs of mice that had been intravenously injected with MDA231-LM2 breast cancer cells, a highly metastatic derivative of MDA-MB-231 (MDA231) cells^10^. ECs were purified from lungs with metastases at different stages for transcriptomic studies (Extended Data Fig. 1a,b). Analysis of the endothelial marker CD31 in metastatic nodules at week 1, 2 or 3 post cancer cell injection revealed that, although early nodules (week 1 or 2) grew in proximity to blood vessels, the presence of vessels within metastatic nodules was primarily observed at a later stage (week 3) (Fig. 1a–c). Numbers of ECs also correlated with numbers of cancer cells in the lung, suggesting active EC proliferation in growing metastases (Fig. 1d).

To purify ECs from lungs with metastases, we used fluorescence-activated cell sorting (FACS) to exclude cells expressing hematopoietic, epithelial, fibroblastic or cancer cell markers (CD45, CD11b, EpCAM, CD140a/b and green fluorescent protein (GFP)), and to select cells expressing the endothelial marker CD31 (Fig. 1e and Extended Data Fig. 1c). Purity of selected populations was determined by expression of additional endothelial markers or distinct markers of other stromal cells (Extended Data Fig. 1d). Isolated ECs were subjected to transcriptomic analysis using microarrays. Principal component analysis (PCA) revealed a notable pattern of gene expression changes at different time points. Certain changes occurred at weeks 1 and 2, but marked differences were not observed between the two time points; however, the most striking changes occurred at week 3, distinguishing this time point from the others (Fig. 1f). Further analyses, such as gene set enrichment analysis (GSEA), Gene Ontology (GO) term analysis or *z*-score analysis, revealed pronounced induction of gene signatures associated with cell proliferation or inflammation at week 3 (Fig. 1g, Extended Data Fig. 1e–g and Supplementary Table 1). Consistent with these findings, angiogenic activity^11,12^ was promoted in ECs at week 3 and vascular changes linked to increased permeability (REACTOME) were also observed (Fig. 1h and Extended Data Fig. 1h). In line with changes in EC proliferation and inflammation, specific targets of transcription factors or pathways involved in these processes were upregulated at week 3 (Extended Data Fig. 1i and Supplementary Table 2). To address the significance of these EC functions in human metastases, we assembled genes involved in proliferation or inflammation that were particularly upregulated in ECs from metastases (Supplementary Tables 3 and 4). We applied these specific metastasis-associated EC signatures to transcriptomic datasets from dissected human metastases and observed that both signatures were associated with poor outcome in breast cancer patients with lung metastases (Extended Data Fig. 1j). Moreover, gene signatures associated with poor clinical outcome^13,14^ were induced in ECs at week 3, suggesting that endothelial activation is linked to metastatic progression (Fig. 1i). Focusing on mediators of intercellular communications, we analyzed the expression of extracellular proteins (GO:0005576) in metastasis-associated ECs. We found significant changes in 58 genes of secreted proteins (GSP58) that were particularly induced in ECs at week 3 (Fig. 1j and Supplementary Table 5). Notably, GSEA or Kaplan–Meier analysis showed that high expression of GSP58 in metastases was associated with poor lung metastasis-free survival in patients with breast cancer, implying that lung ECs may promote metastasis via some of these secreted factors (Fig. 1k and Extended Data Fig. 1k,l).

### GSP58 expression in ECs is largely independent of VEGF signaling

**Fig. 2:**
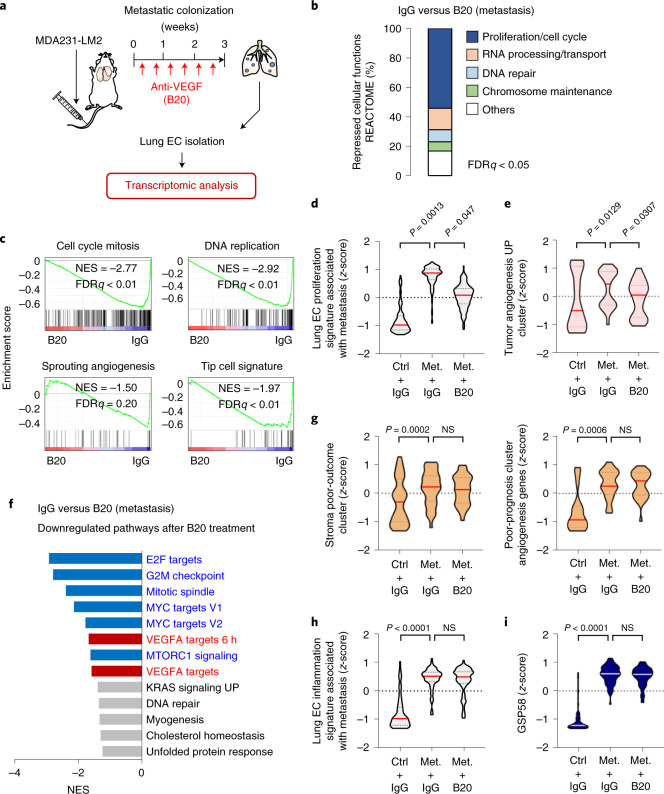
Anti-VEGF treatment inhibits proliferation, but not inflammatory responses or induction of GSP58 signature, in metastasis-associated ECs. **a**, Experimental outline of anti-VEGF treatment of mice with metastatic lung nodules. MDA231-LM2 cells were injected intravenously into NSG mice followed by repeated treatment with either anti-VEGF antibody B20.4.1.1 (B20) or control IgG for 3 weeks. Lung ECs were isolated at week 3 and transcriptomic analysis was performed. Mice harboring comparable lung metastatic loads, as measured by in vivo bioluminescence imaging, were selected for analysis. **b**, Overview of cell function signatures (REACTOME) repressed in metastasis-associated ECs by anti-VEGF treatment. Signatures with FDR < 0.05 were included in calculations of percentages. **c**, GSEA of gene clusters: cell cycle mitosis (REACTOME), DNA replication (REACTOME), sprouting angiogenesis (C5 collection in MSigDB) and tip cell signature^12^ in lung ECs after B20 treatment compared with control IgG. FDR was determined from *P* values calculated by random permutation test. **d**,**e**, Violin plots showing *z*-scores of genes from a lung EC proliferation signature associated with metastasis (Supplementary Table 3) (**d**) and tumor angiogenesis UP cluster^16^ (**e**) expressed in purified lung ECs from control (ctrl) or metastasis (met.)-bearing mice with the indicated treatment. **f**, Downregulated gene signatures in metastasis-associated ECs treated with B20 compared to IgG control, based on GSEA. Signatures with FDR < 0.25 are shown. Blue, proliferation-related signatures; red, signatures of VEGF target genes; gray, others. **g**–**i**, Violin plot analysis of stroma poor-outcome cluster^13^ (**g**, left) and angiogenesis genes associated with poor prognosis^14^ (**g**, right); lung EC inflammation signature associated with metastasis (Supplementary Table 4) (**h**) and GSP58 (**i**) expressed in ECs from mouse lungs under the indicated conditions. In **d**,**e** and **g**–**i**, *P* values were determined from averaged *z*-score of genes within signatures by one-way ANOVA with Dunnett’s multiple comparison test; *n* = 3 mice per group. NS, not significant. Source data

**Extended Data Fig. 2:**
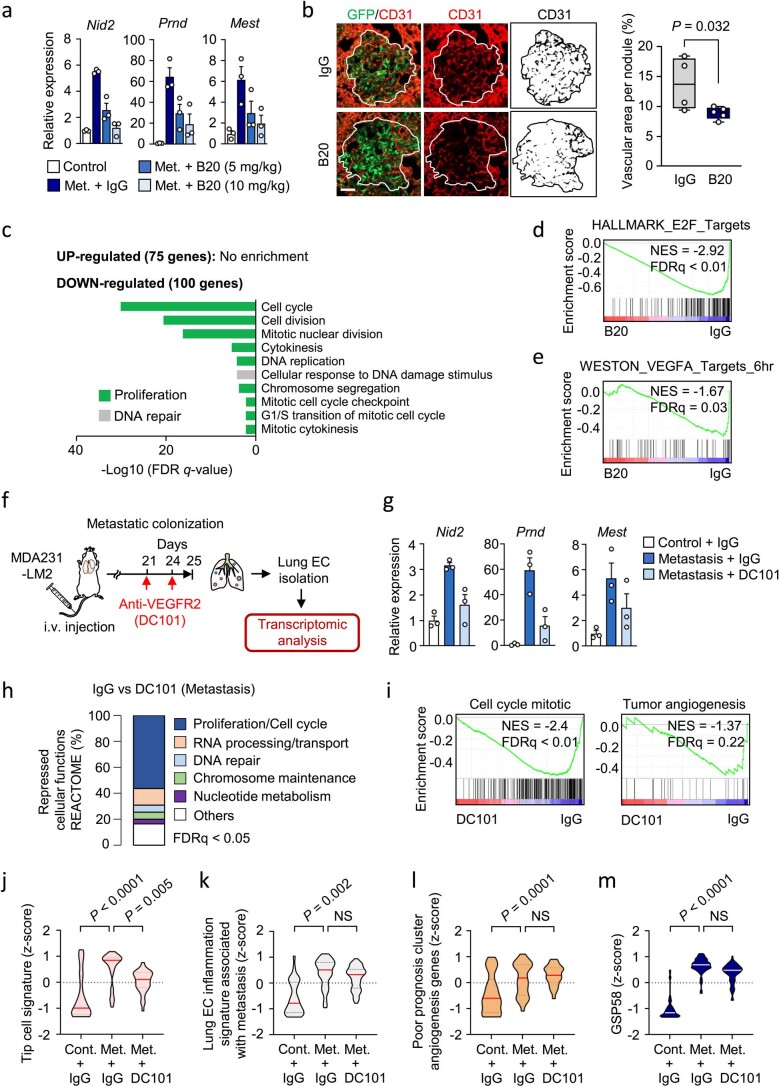
Inhibition of VEGF signaling in ECs during lung metastasis. **a**, Expression of VEGF target genes in lung ECs isolated from healthy control mice or mice with lung metastasis (at week 3) treated with IgG or anti-VEGFA antibody (B20); *n*= 3 experiments. Means with s.e.m. are shown. **b**, Immunofluorescence analysis of ECs (CD31, red) in metastatic nodules in lungs at week 3 from mice treated with control IgG or B20 antibodies. MDA231-LM2 cancer cells are green (GFP). Representative images (left) and quantification of vascular area per nodule (right). Scale bar, 50 μm. *P* value was determined by one-tailed Mann-Whitney test; *n*= 4 mice (IgG) and 5 mice (B20). Boxes show median with upper and lower quartiles and whiskers indicate maximum and minimum values. **c**, GO analysis of differentially regulated genes (*P*< 0.05, log2FC> ± 0.5) in lung ECs following *in vivo* treatment with B20. Shown are biological processes enriched with FDR< 0.05. **d**,**e**, GSEA of “E2F targets” (Hallmark in MSigDB) and “VEGF targets”^17^ comparing lung ECs from mice treated with IgG or B20. NES, normalized enrichment score. **f**, Experimental outline for the analysis of VEGFR2 function in metastasis-associated lung ECs. MDA231-LM2 cells were injected intravenously followed by treatment with anti-VEGFR2 antibody (DC101), or control IgG. ECs were isolated from metastatic lungs and transcriptomic analysis performed. Mice harboring comparable metastatic loads in lungs, measured by *in vivo* bioluminescence imaging, were selected for analysis. **g**, Expression of VEGF target genes in lung ECs isolated from healthy control mice or mice with lung metastasis treated with IgG or DC101. Data are means with s.e.m. from 3 experiments. **h**, Overview of REACTOME pathway genes down-regulated by DC101. Pathways with FDR< 0.05 are shown. **i**, Repression of gene signatures of “Cell cycle mitotic” (REACTOME) and “Tumor angiogenesis” (tumor angiogenesis UP cluster)^16^ in lung ECs treated as in panel f. NES, normalized enrichment score. **j-m**, Violin plots showing z-scores of genes from indicated signatures expressed in ECs from lungs of mice under indicated conditions. Signatures: tip cells^12^, lung EC inflammation associated with metastasis (Supplementary Table 4), poor prognosis angiogenesis genes^14^ and GSP58. *P* values were determined by one-way ANOVA with Dunnett’s multiple comparison test; *n*= 3 for each group. NS, not significant. Source data

Considering that VEGF is a key regulator of EC biology and angiogenesis, we asked whether the gene expression changes in metastasis-associated ECs were dependent on VEGF. We treated metastases-bearing mice with either anti-VEGF antibody (B20.4.1.1)^15^ or isotype IgG and isolated lung ECs for transcriptomic analysis (Fig. 2a). Specific VEGF target genes were repressed in lung ECs and vascular growth was significantly reduced in metastatic lungs, indicating a robust inhibition of VEGF signaling (Extended Data Fig. 2a,b). Notably, gene signatures linked to cellular proliferation and angiogenesis^16,17^ were also repressed in lung ECs from anti-VEGF-treated mice (Fig. 2b–f and Extended Data Fig. 2c–e). However, despite repression of VEGF signaling and EC proliferation, gene clusters linked to poor patient outcome were not affected by anti-VEGF treatment (Fig. 2g). Importantly, inflammatory responses and GSP58 were also generally unaffected by anti-VEGF, indicating a VEGF-independent regulation (Fig. 2h,i). Concordant results were observed when we analyzed lung ECs from mice treated with an antibody targeting VEGF receptor 2 (anti-VEGFR2, DC101) (Extended Data Fig. 2f). Whereas both expression of VEGF target genes and proliferation and angiogenesis signatures were repressed in lung ECs by anti-VEGFR2 treatment, inflammation and poor-outcome gene clusters or GSP58 were not significantly affected (Extended Data Fig. 2g–m). These results indicate that, although anti-VEGF treatment effectively suppresses vascularity within metastatic nodules, it does not repress expression of most of the 58 factors, secreted by the vasculature, that were associated with poor outcome in patients.

### ECs express genes of secreted factors that promote metastasis

**Fig. 3:**
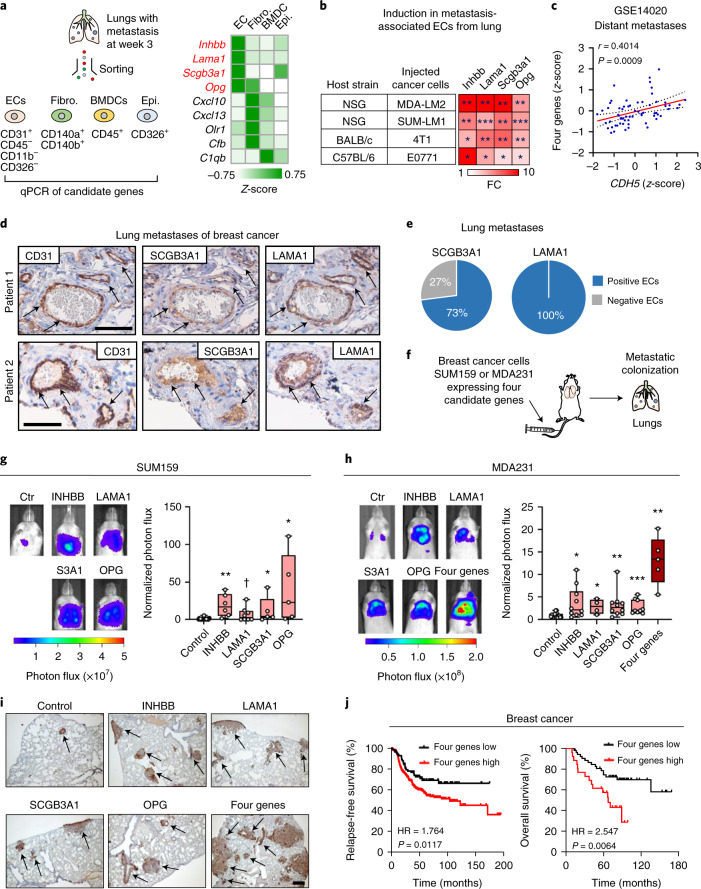
Secreted factors produced by reactive ECs promote lung metastasis. **a**, Analysis of endothelial niche candidate genes in different stromal cells isolated from metastatic lungs. Heatmap summarizes expression of genes with log_2_FC > 3.5. *Z*-scores were calculated from qPCR analysis of different stromal cells isolated from three or four mice per group. Genes highly expressed in ECs are marked in red. BMDC, bone marrow-derived cell; fibro., fibroblasts; epi., epithelial cells. **b**, Expression of four endothelial niche candidate genes in lung ECs isolated from indicated mouse strains with metastasis of different breast cancer cell lines. Heatmap was generated from qPCR analyses and *P* values calculated by unpaired one-tailed *t*-test. *P < 0.05, **P < 0.01, ***P < 0.001; *n* = 3–4 mice per group. **c**, Correlation analysis of expression of the four candidate genes and *CDH5* in metastatic lesions of patients with breast cancer (GSE14020). Linear regression with Pearson correlation (*r*) and two-tailed *P* value is shown; *n* = 65. **d**, Immunohistochemical analysis of CD31, SCGB3A1 and LAMA1 expression in lung metastasis samples from patients with breast cancer; shown are two representative examples of metastases from analysis of 11 patients. Arrows indicate positively stained endothelial cells. Scale bars, 100 μm (patient 1) and 60 μm (patient 2). **e**, Proportion of lung metastasis samples expressing SCGB3A1 or LAMA1. **f**–**h**, Metastatic colonization of lungs (**f**) by SUM159 breast cancer cells (**g**) and MDA231 cells (**h**) overexpressing endothelial niche factors or a control vector. **g**,**h**, Bioluminescence images (left) and normalized photon flux (right) 42 days post intravenous injection by SUM159 (**g**) and MDA231 (**h**) breast cancer cells. S3A1, SCGB3A1. Boxes depict median with upper and lower quartiles. Data points show values of biological replicates and whiskers indicate minimum and maximum. **g**, *P* values were calculated by one-tailed Mann–Whitney test. *P < 0.05, **P < 0.01, ***P < 0.001,^✝^*P* = 0.069. Control, *n* = 20; INHBB, *n* = 6; LAMA1, *n* = 8; SCGB3A1, *n* = 5; OPG, *n* = 5. **h**, Control, *n* = 19; INHBB, *n* = 10; LAMA1, *n* = 4; SCGB3A1, *n* = 10; OPG; *n* = 9; and four genes, *n* = 5. **i**, Histological examples of metastases marked by expression of human vimentin in lungs of mice from **h**. Shown are representatives from at least four independent samples. Scale bar, 200 μm. **j**, Kaplan–Meier analyses on compiled datasets of ER^–^ breast cancers (KM plotter) examining association of the four endothelial niche factors with relapse-free survival (*n* = 347 patients) and overall survival (*n* = 79 patients). *P* values were determined by log-rank test. HR, hazard ratio. Source data

**Extended Data Fig. 3:**
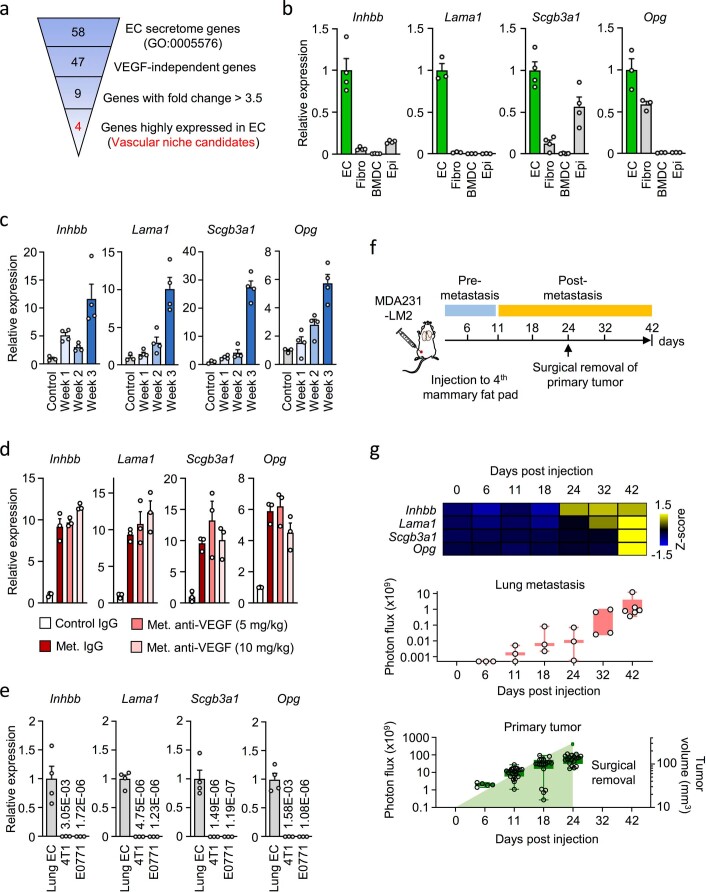
Expression of vascular niche components in lung metastasis. **a**, Overview of the process used to narrow down gene candidates of the pro-metastatic vascular niche in lungs. **b**, Relative expression of four vascular niche factors in lung ECs, fibroblasts (Fibro), bone marrow-derived cells (BMDC) and epithelial cells (Epi) isolated from lungs harboring metastasis (week 3), as in Fig. 3a. Data are means with s.e.m. from 3 mice (*Lama1* and *Opg*) or 4 mice (*Inhbb* and *Scgb3a1*) per group. **c**, Expression kinetics of niche components during metastatic colonization of lungs. Means with s.e.m. from 3 mice (control) or 4 mice (metastasis week 1, 2 and 3) for each group are shown. **d**, Relative expression of niche factors in ECs isolated from lungs of healthy control mice or mice harboring metastasis and treated with IgG or anti-VEGF antibody (B20). Data are means with s.e.m. from 3 mice per group. **e**, Relative expression of the niche factors in lung ECs (*n*= 4 mice) and the mouse mammary tumor cells 4T1 and E0771 (*n*= 3). Means with s.e.m. are shown. **f**, Experimental setup to analyze kinetics of vascular niche activation. **g**, Expression kinetics of four vascular niche factors in lung ECs in context of spontaneous metastasis from mammary tumors to lungs. Shown are z-scores based on qPCR (top), metastatic burden in mouse lungs at different time points based on *ex vivo* bioluminescence (middle, *n*= 3 lungs for day 6, 11, 18, 24; *n*= 4 lungs for day 32 and *n*= 6 lungs for day 42 post injection) and primary tumor growth measured by bioluminescence (bottom, *n*= 5 for day 6, *n*= 23 for day 11, *n*= 19 for day 18, and *n*= 18 for day 24 post injection) or by caliper (mm^3^, bottom, *n*= 18 for day 18, and *n*= 17 for day 24 post injection). Boxes show median with upper and lower quartiles and whiskers indicate maximum and minimum values. Source data

To identify gene candidates from GSP58 for which ECs serve as a primary cellular source compared with other stromal cells, we investigated those genes most highly induced within the signature. All VEGF-independent genes within GSP58 that were induced 3.5-fold or more were selected for further analysis, and their expression determined in four different stromal cell types isolated from mouse lung harboring metastases (Fig. 3a and Extended Data Fig. 3a). Expression analysis in lung ECs, fibroblasts, hematopoietic cells and epithelial cells showed that four genes—that is, inhibin subunit beta B (*Inhbb*), laminin subunit alpha 1 (*Lama1*), secretoglobin family 3A member 1 (*Scgb3a1*) and TNF receptor superfamily member 11b, also called osteoprotegerin (*Opg*)—were distinctly expressed in ECs compared with other cell types (Fig. 3a and Extended Data Fig. 3b).

INHBB is a protein subunit of activins and inhibins, members of the TGF-β family, of which activins have been shown to regulate wound healing, fibrosis and cancer^18^. *LAMA1* encodes the α-chain of laminin trimers LN111 and LN121, which are broadly expressed during embryonic development but more restricted in adult tissues^19^. Notably, LN111 is a component of certain basement membranes. SCGB3A1 is a member of the secretoglobin family of small secreted molecules and is expressed in normal lung, but its function is poorly understood^20^. OPG, a member of the TNF receptor superfamily, is generally recognized to have a pleiotropic function but its best characterized role is acting as decoy receptor of TRAIL or RANKL^21^.

**Extended Data Fig. 4:**
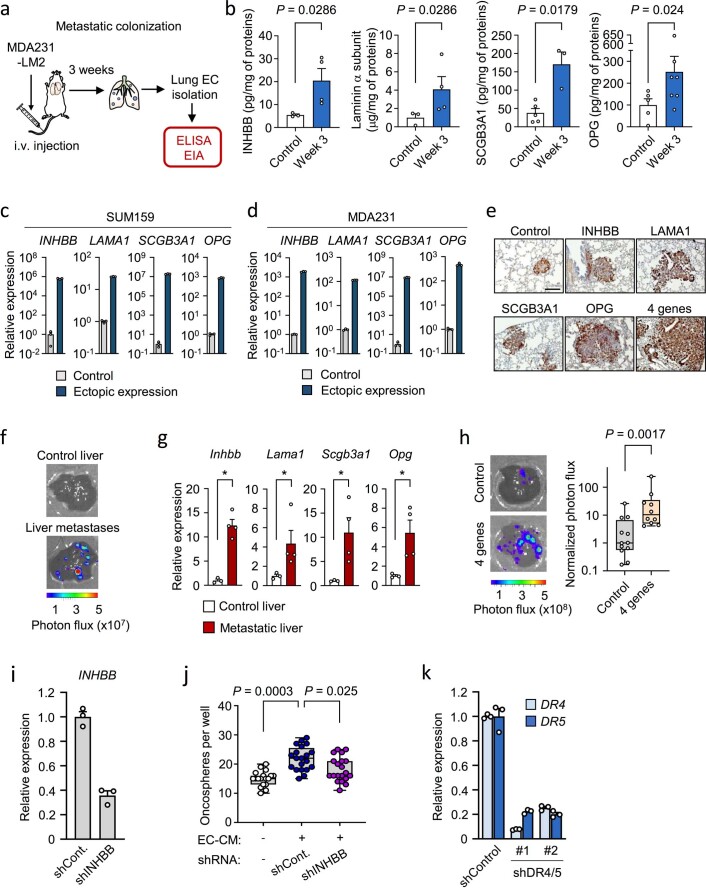
Functional analysis of vascular niche factors in lung and liver metastasis. **a**, Experimental setup to analyze protein levels of the four niche components in metastasis-associated ECs. **b**, Protein levels of vascular niche factors analyzed by ELISAs on ECs from mouse lungs harboring metastases compared to control lungs, *n*= 3 (control) and 4 (week 3) for INHBB or Laminin α subunit, *n*= 5 (control) and 3 (week 3) for SCGB3A1, and *n*= 5 (control) and 7 (week 3) for OPG. Data are means with s.e.m., and *P* values were determined by one-tailed Mann-Whitney test. **c,d**, Expression of vascular niche factors in SUM159 or MDA231 cancer cells overexpressing cDNA of each gene. **e**, High magnification of images showing immunohistochemical analysis of vimentin expression in lung metastases from Fig. 3i. Shown are representative examples from minimum 4 independent samples. Scale bar 100 μm. **f**, Metastases in liver. Representative examples of bioluminescence from luciferase transduced cancer cells in control liver and liver with metastasis. **g**, Expression of four niche components in ECs isolated from livers with metastases; *n*= 3 (control), *n*= 4 (metastasis). Means with s.e.m. are shown. *P* values were determined by one-tailed Mann-Whitney test. *P< 0.05. **h**, Metastases in liver of mice implanted with breast cancer cells ectopically expressing the four niche components. Left, representative liver bioluminescence. Right, quantification of liver metastasis based on *ex vivo* bioluminescence. *P* values were determined by one-tailed Mann-Whitney test; *n*= 12 (control) and *n*= 10 (4 gene expression). **i**, INHBB expression in ST1.6R ECs transduced with shControl or shINHBB. **j**, Oncosphere formation of SUM159-LM1 cultured with CM from shControl or shINHBB ST1.6R cells. Data shown are all technical replicates (CM -, *n*= 19; CM +/shControl, *n*= 20; CM +/shINHBB, *n*= 20) from 5 independent experiments and *P* values were determined by one-way ANOVA with Dunnett’s multiple comparison test. **k**, Expression of TRAIL activated death receptors 4/5 (DR4/5) in MDA231 cancer cells transduced with shControl or shDR4/5 (#1 and #2 are independent hairpins). Means with s.e.m. of 3 technical replicates from qPCR results are shown in c, d, i and k. Boxes in h and j indicate medians with upper and lower quartiles and whiskers represent maximum and minimum values. Source data

We observed consistent induction of *Inhbb*, *Lama1*, *Scgb3a1* and *Opg* in lung ECs from two xenograft models (MDA231-LM2/SUM159-LM1) and two syngeneic mouse models of lung metastasis (4T1/E0771), and confirmed that expression in ECs was unaffected by anti-VEGF treatment (Fig. 3b and Extended Data Fig. 3c,d). These four candidates were not markedly expressed in 4T1 or E0771 cancer cells compared with lung ECs from mice with lung metastasis (Extended Data Fig. 3e). To study these genes in the context of a complete metastatic process, we analyzed ECs from lungs of mice with spontaneous metastasis from mammary glands and observed upregulation of all four candidates (Extended Data Fig. 3f,g). This upregulation was associated with growth of metastases and was not detectable at early time points, even though pronounced primary tumor growth was evident. Notably, induction of the factors in metastasis-associated ECs was not affected by primary tumor removal (Extended Data Fig. 3f,g). To examine protein levels of the niche candidates, we performed enzyme-linked immunosorbent assay (ELISA) or enzyme immunoassay (EIA) on sorted ECs from metastatic lungs and revealed a significant upregulation of all four proteins (Extended Data Fig. 4a,b). To determine the relevance of these niche candidates to human metastases, we analyzed samples of lung metastasis from patients with breast cancer. In these samples, expression of the four candidate genes correlated significantly with the endothelial maker *CDH5* (VE-cadherin) (Fig. 3c). We also analyzed the expression of SCGB3A1 and LAMA1 using immunohistochemistry on tissue sections from 11 human metastasis samples, and observed EC expression of SCGB3A1 in 8/11 samples (73%) and of LAMA1 in 11/11 samples (100%) (Fig. 3d,e and Supplementary Table 6). This encouraged us to study the function of the four candidate genes in metastasis, and thus we ectopically expressed *INHBB*, *LAMA1*, *SCGB3A1 and OPG* individually in human breast cancer cells and injected these intravenously into NSG mice. We expressed the genes in SUM159 and MDA231 cancer cells, the parental lines of SUM159-LM1 and MDA231-LM2, respectively, and observed that all four candidate genes individually promoted metastatic colonization of the lung (Fig. 3f–i and Extended Data Fig. 4c–e). Moreover, combined expression of the four genes in MDA231 cancer cells showed additive induction of metastatic growth in lungs (Fig. 3h,i). This suggested that, as individual genes, *INHBB*, *LAMA1*, *SCGB3A1* and *OPG* can indeed promote metastatic colonization, and that coexpression of the four genes may provide a further advantage to cancer cells. The results indicate that proteins encoded by the four genes are functional components of a pro-metastatic vascular niche.

Patients with breast cancer who are diagnosed with metastatic relapse frequently present with multiple metastases that can seed each other, and such interorgan seeding has been observed between lung and liver^22^. This spiked our interest to analyze potential liver metastasis in mice with late-stage lung metastasis. Bioluminescence analysis of mice with lung metastasis revealed colonization of the liver (Extended Data Fig. 4f). We isolated ECs from liver areas with high bioluminescence and observed that *Inhbb*, *Lama1*, *Scgb3a1* and *Opg* were highly expressed in ECs associated with liver metastasis compared with controls (Extended Data Fig. 4g). Importantly, ectopic expression of the vascular niche factors promoted metastasis of the liver (Extended Data Fig. 4h). This suggests a functional role of the four niche components that extends beyond lung metastasis and to other metastatic sites such as the liver.

Finally, to address the potential association of vascular niches with clinical outcome, we performed Kaplan–Meier analyses using expression of the four vascular niche components in estrogen receptor (ER)-negative breast cancer samples and investigated a potential link to survival. In these samples, expression of the vascular niche factors was significantly associated with poor relapse-free and overall survival, indicating a potential role in breast cancer (Fig. 3j).

### INHBB and SCGB3A1 induce stem cell properties in breast cancer cells

**Fig. 4:**
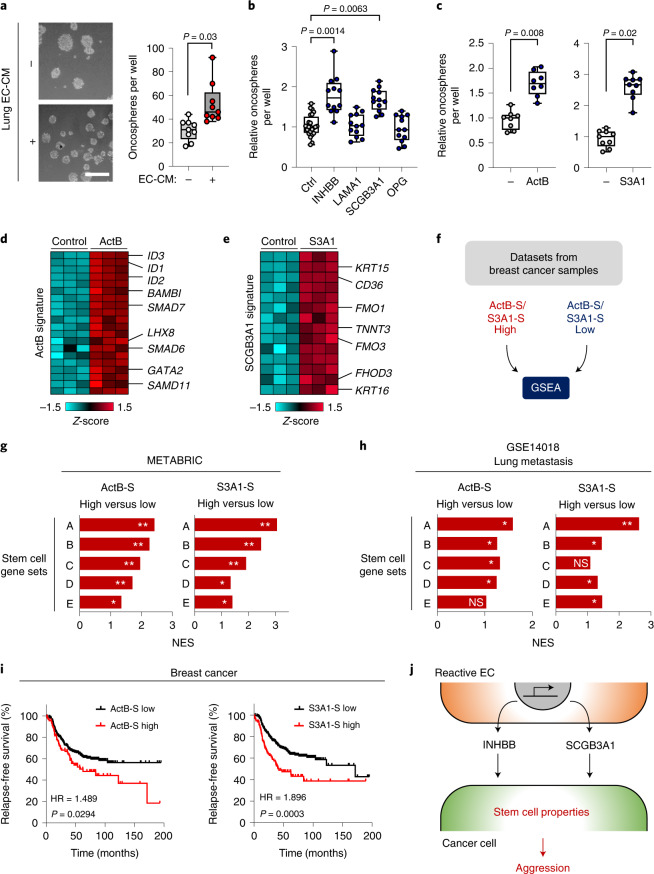
The INHBB homodimer, activin B, and SCGB3A1 promote stem cell properties of breast cancer cells. **a**, Oncosphere formation of SUM159-LM1 breast cancer cells cultured with CM from ST1.6R human lung ECs. CM^–^, *n* = 9; CM^+^, *n* = 9 technical replicates from 3 independent experiments. Scale bar, 250 μm. **b**, Analysis of oncosphere formation in SUM159-LM1 breast cancer cells stimulated with HEK293-derived CM containing indicated factors of the vascular niche. Control, *n* = 24; INHBB, *n* = 12; LAMA1, *n* = 12; SCGB3A1, *n* = 12; OPG, *n* = 12 technical replicates from 4 independent experiments. **c**, Oncosphere formation in SUM159-LM1 stimulated with either recombinant activin B (ActB, 50 ng ml^–1^) or SCGB3A1 (S3A1, 1 μg ml^–1^). ActB^–^, *n* = 8; ActB^+^, *n* = 8; S3A1^–^, *n* = 9; S3A1^+^, *n* = 9 technical replicates from 4 (ActB) or 3 (S3A1) independent experiments. **a**–**c**, *P* values were determined by ratio-paired, two-tailed *t*-test from biologically independent experiments. Boxes depict median with upper and lower quartiles and whiskers indicate minimum and maximum values. **d**,**e**, Heatmaps of top upregulated genes in response to ActB (**d**) or S3A1 (**e**). **f**, Schematic diagram of GSEA setup to analyze datasets from patients with breast cancer stratified according to expression of ActB or S3A1 signatures (ActB-S or S3A1-S). **g**,**h**, Stem cell-associated gene sets enriched in patients with high ActB-S or S3A1-S in breast cancer samples from Metabric (**g**) or, GSE14018 (**h**) datasets. Stem cell signatures are indicated as follows: A, Lim_Mammary stem cell up; B, Lee_Neural crest stem cell up; C, Oswald_Hematopoietic stem cell in collagen gel up; D, Yamashita_Liver cancer stem cell up; E, Ivanova_Hematopoiesis stem cell (all from C2 collection in MSigDB). Dataset from triple-negative breast cancer samples in METABRIC discovery (upper and lower quantile, *n* = 66) and lung metastases of breast cancer in GSE14018 (upper and lower quantile, *n* = 8) were analyzed. FDR was determined from *P* values calculated by random permutation test. *FDR < 0.25, **FDR < 0.05. **i**, Kaplan–Meier analysis of relapse-free survival of ER^–^ patients with breast cancer stratified according to expression of ActB-S or S3A1-S; *n* = 347 patients. *P* values were determined by log-rank test. **j**, Diagram summarizing the findings on roles of INHBB and SCGB3A1 in breast cancer metastasis. Source data

Vascular niches have been recognized to produce secreted factors that support stem cell properties of certain malignancies such as brain tumors^23^. To address this in the context of metastatic breast cancer, we studied the potential of conditioned medium (CM) from ECs to stimulate cancer cells to form spheres on ultra-low adhesive plates, a method that promotes stem cell attributes^24,25^. EC-CM significantly enhanced sphere formation by breast cancer cells (Fig. 4a). Therefore, we wanted to address the potential of each of the four vascular niche components to promote sphere formation. SUM159-LM1 breast cancer cells were treated with CM from HEK293T cells, containing individual niche factor, and oncosphere formation was determined. The sphere-forming ability of breast cancer cells was markedly increased when treated with CM containing INHBB or SCGB3A1, but not with OPG or LAMA1 (Fig. 4b). Considering these results, we stimulated SUM159-LM1 breast cancer cells with either recombinant activin B, a homodimer of INHBB, or recombinant SCGB3A1, and this also induced sphere formation by cancer cells (Fig. 4c). Moreover, CM obtained from lung ECs transduced with short hairpin RNA against *INHBB* showed reduced potential to stimulate oncospheres compared with control CM (Extended Data Fig. 4i,j). The results suggest a putative role for INHBB and SCGB3A1 in the promotion of stem cell properties in breast cancer cells. To explore this connection further, we performed transcriptomic analysis on SUM159 breast cancer cells treated with activin B or SCGB3A1 and revealed their respective response signatures (Fig. 4d,e and Supplementary Tables 7 and 8). Notably, within the activin B signature, three members of the family of inhibitors of DNA binding and cell differentiation (ID) proteins (ID1, ID2 and ID3) were among the top induced genes (Fig. 4d). These proteins are recognized to promote stem cell self-renewal and multipotency, and were previously shown to facilitate metastatic colonization by breast cancer cells^26,27^.

To investigate the potential stem cell phenotype associated with activin B or SCGB3A1 signatures in clinical samples, we stratified gene expression datasets from breast cancer patients (METABRIC or lung metastasis samples) based on expression of activin B or SCGB3A1 signatures (Fig. 4f and Supplementary Tables 7 and 8). GSEA revealed that patients with high activin B- or SCGB3A1-mediated gene responses exhibited enrichment in several stem cell signatures (Fig. 4g,h). Importantly, Kaplan–Meier analysis showed that activin B and SCGB3A1 signatures were both associated with poor clinical outcome in patients with breast cancer (Fig. 4i). Together, these results suggest that INHBB and SCGBA1 promote stem cell properties and aggression in breast cancer cells (Fig. 4j).

### OPG and LAMA1 support viability of metastatic breast cancer cells

**Fig. 5:**
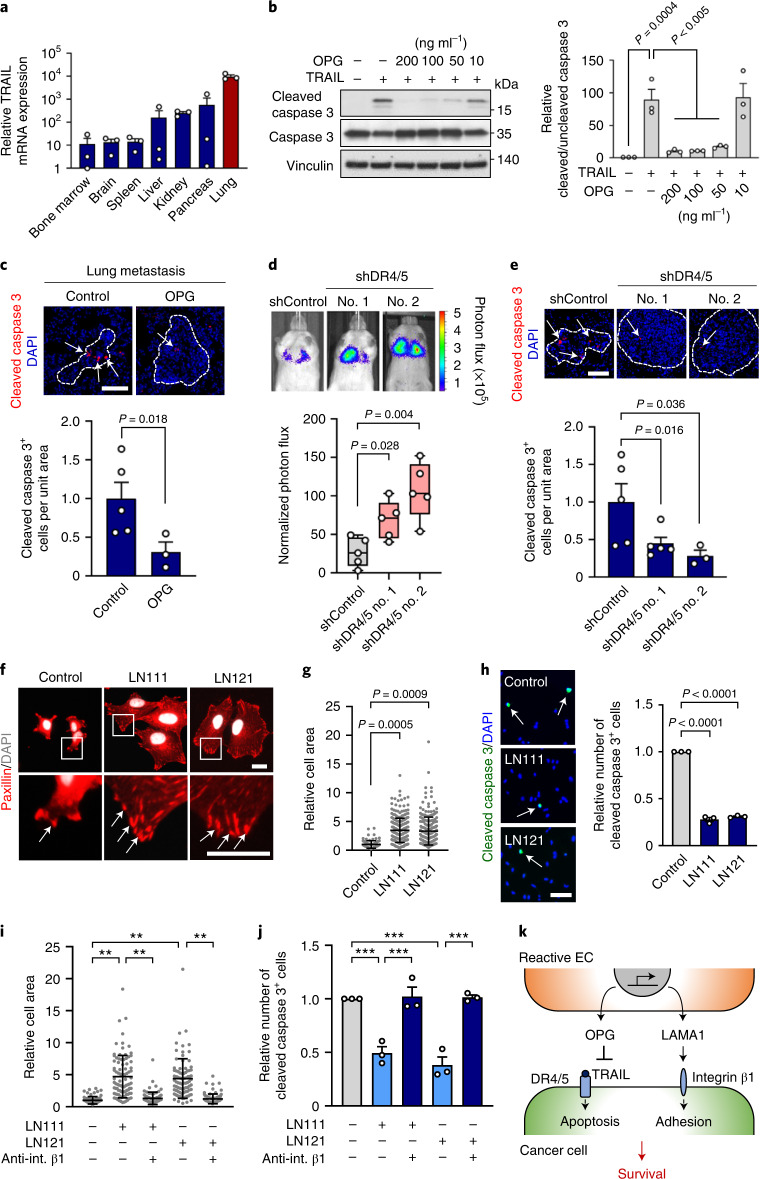
OPG and LAMA1 regulate breast cancer cell survival. **a**, TRAIL messenger RNA levels in different organ tissues isolated from NSG mice; *n* =3 mice per group. Expression was determined by qPCR. Data are means with s.e.m. **b**, Left, immunoblot analysis of cleaved caspase 3 expression in MDA231-LM2 cells treated with TRAIL (50 ng ml^–1^) and with indicated concentrations of OPG. Right, quantification based on the ratio between cleaved and uncleaved caspase 3. Means with s.e.m. from three independent experiments are shown. Statistical analysis was performed with one-way ANOVA and Dunnett’s multiple comparison test. **c**, Immunofluorescence analysis of cleaved caspase 3 expression in lung metastasis from mice injected with OPG-expressing MDA231 cancer cells. Top, representative examples; cell nuclei stained with DAPI. Scale bar, 100 μm. Bottom, quantification; *n* = 5 (control) and *n* = 3 (OPG). Values are means with s.e.m. *P* value was determined by one-tailed Mann–Whitney test. **d**, Lung colonization of MDA231 cancer cells transduced with shRNA control (shControl) or shRNA against death receptors 4 and 5 (shDR4/5, two independent hairpins). Representative bioluminescence images (top) and normalized photon flux (bottom) 21 days after intravenous injection are shown; *n* = 5 mice per group. Boxes show median with upper and lower quartiles, and whiskers indicate maximum and minimum. *P* values were calculated by one-tailed Mann–Whitney test. **e**, Cleaved caspase 3 analysis of lung metastasis as in **d**. Top, representative examples; nuclei stained by DAPI. Scale bar, 100 μm. Bottom, quantification; *n* = 5 mice (control and shDR4/5 no. 1) and *n* = 3 mice (shDR4.5 no. 2). Data are means with s.e.m. *P* values were determined by one-tailed Mann–Whitney test. **f**, Immunofluorescence analysis of paxillin expression in breast cancer cells plated on LN111 or LN121. DAPI was used to stain nuclei. Arrows indicate dense paxillin at focal adhesions; *n* = 3. Scale bar, 20 μm. **g**, Analysis of spreading of breast cancer cells plated onto LN111 or LN121. Shown are relative cell areas of all cells examined over three independent experiments, with means and s.d. **h**, Expression of cleaved caspase 3 in breast cancer cells plated on LN111 or LN121 matrix. Data are means with s.e.m. from three independent experiments. Scale bar, 100 μm. **i**,**j**, Integrin β1 function in laminin-induced cell spreading and survival. Relative cell area (**i**) and cleaved caspase 3 expression (**j**) were analyzed in cells plated on LN111 or LN121 matrix with or without neutralizing antibody against integrin β1 (anti-int. β1). Shown are means ± s.d. with relative cell area of all examined cells (**i**) or means with s.e.m. (**j**) from three independent experiments. *P* values were determined by one-way ANOVA with Dunnett’s (**g**,**h**) or Tukey’s (**i**,**j**) multiple comparison test from three independent experiments. **P< 0.01, ***P< 0.001. **k**, Schematic summarizing OPG and LAMA1 functions in breast cancer metastasis. Source data

Prompted by OPG potential to function as a decoy receptor for TRAIL^21^, we analyzed its function in the context of lung metastasis. TRAIL is highly expressed in the lung microenvironment (Fig. 5a) and is an important regulator of apoptosis in the lung during metastasis^28^. We analyzed TRAIL-induced apoptosis in MDA231-LM2 breast cancer cells by cleaved caspase 3 expression in the presence of incremental levels of OPG, and observed OPG-mediated protection from TRAIL (Fig. 5b). This suggested that OPG, induced in metastasis-associated ECs, may protect invading cancer cells from TRAIL-induced apoptosis. Indeed, we observed reduced apoptosis in metastases overexpressing OPG compared to control metastases in mice (Fig. 5c). TRAIL-induced apoptosis is mediated by death receptors 4 and 5 (DR4/5)^29,30^. To address the significance of these receptors for metastatic colonization of the lung, we induced shRNA-mediated knockdown of DR4/5 in MDA231 breast cancer cells (Extended Data Fig. 4k) and injected these intravenously into NSG mice. DR4/5 knockdown enhanced metastatic colonization of the lung (Fig. 5d) and less apoptosis was observed in lung nodules (Fig. 5e), suggesting that this mechanism plays a role in protection of the lung against metastatic colonization.

To analyze the functional role of LAMA1, we plated breast cancer cells onto surfaces coated with LN111 or LN121, the only laminin trimers containing the α-chain transcribed by LAMA1. This resulted in enhanced focal adhesion, based on increased expression and punctuated localization of paxillin with increased cellular spreading (Fig. 5f,g). Moreover, analysis of cleaved caspase 3 expression in breast cancer cells under serum starvation revealed that LN111 and LN121 can inhibit apoptosis (Fig. 5h). Notably, LN111- and LN121-induced spreading and resistance to apoptosis were dependent on the adhesion receptor integrin β1 expressed by breast cancer cells (Fig. 5i,j). Taken together, the results on OPG and LAMA1 function suggest that they respectively promote protection from TRAIL-induced apoptosis and adhesion-mediated survival of breast cancer cells (Fig. 5k).

### The vascular niche is regulated by macrophages in metastatic lung

**Fig. 6:**
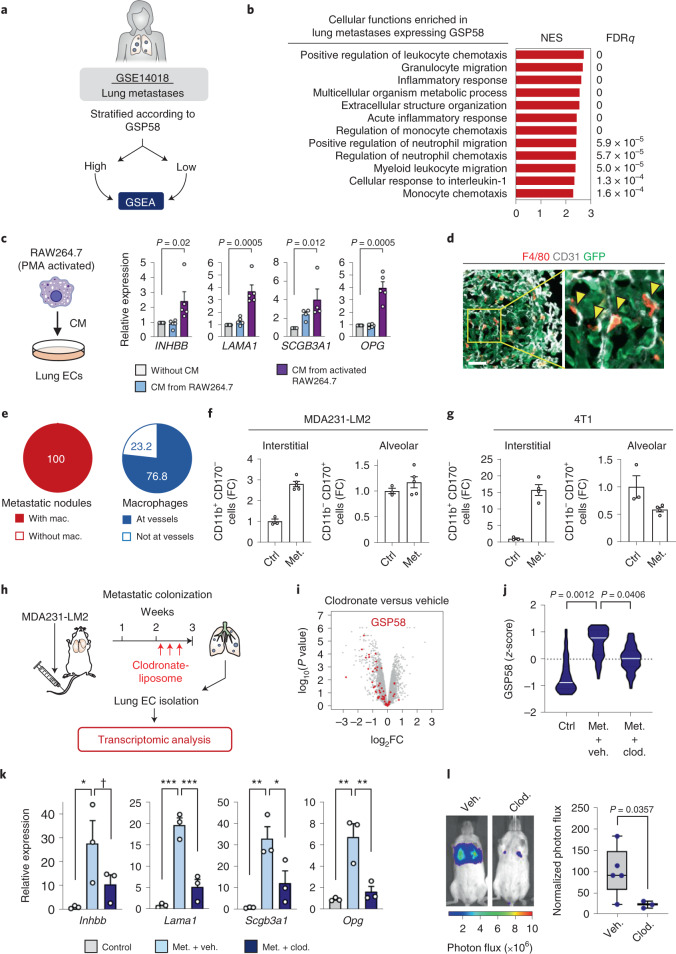
The endothelial niche is regulated by perivascular macrophages in lung metastasis. **a**, Schematic overview of GSEA setup used to analyze datasets from human metastasis samples ranked according to GSP58. All patients with lung metastasis (16 patients) were selected from GSE14018. **b**, Cellular functions enriched in GSP58-expressing human lung metastases. Cellular functions are ranked based on NES. FDR was determined from *P* values calculated by random permutation tests. **c**, Expression of niche factors in ECs treated with CM from naïve or activated macrophage cell line RAW264.7. Means with s.e.m. from either four (*SCGB3A1*) or five (*INHBB*, *LAMA1* and *OPG*) independent experiments are shown. Statistical analysis was performed with ratio-paired, two-tailed *t*-tests. **d**, Immunofluorescence analysis of endothelial cells (CD31, white), macrophages (F4/80, red) and MDA231-LM2 (GFP, green) in metastatic nodules from mouse lung. Shown are representative images from four independent samples. Arrowheads indicate perivascular localization of macrophages. Scale bar, 50 μm. **e**, Quantification (percentages) of nodules with infiltrated macrophages (mac.) or macrophages associated with vessels. **f**,**g**, Macrophage subpopulations within lungs of healthy mice or mice harboring lung metastases, analyzed by flow cytometry. Interstitial and alveolar macrophages were quantified in MDA231-LM2- (**f**) and 4T1-mediated (**g**) lung metastases in NSG or BALB/c mice, respectively; *n* = 3 (control), *n* = 5 (metastasis, **f**) and *n* = 4 (metastasis, **g**). Means with s.e.m. are shown. **h**, Experimental setup of clodronate (clod.)-mediated macrophage depletion in mice with lung metastasis, followed by transcriptional analysis of lung ECs. **i**, Volcano plot showing differential expression of genes in lung ECs after macrophage depletion (GSP58 highlighted in red). **j**, Violin plot showing expression of GSP58 in ECs from lungs of metastasis-bearing mice following macrophage depletion. *P* values were determined by one-way ANOVA with Dunnett’s multiple comparison test; *n* = 3 for each group. **k**, Expression of endothelial niche factors in purified lung ECs as in **h**–**j**. mRNA levels were determined by qPCR; *n* = 3 mice. Shown are means with s.e.m., and *P* values were calculated by one-way ANOVA with Holm–Sidak’s multiple comparison test. *P < 0.05, **P < 0.01, ***P < 0.001, ^✝^*P* = 0.086. **l**, Lung colonization of MDA231-LM2 cells in mice after clodronate-liposome treatment. Representative bioluminescence (left) and normalized photon flux at day 14 (right); *n* = 5 mice (vehicle (veh.) and PBS-liposome) and *n* = 3 mice (clodronate-liposome). Boxes show medians with upper and lower quartiles, and whiskers represent minimum and maximum values. *P* values were calculated by one-tailed Mann–Whitney test. Source data

**Extended Data Fig. 5:**
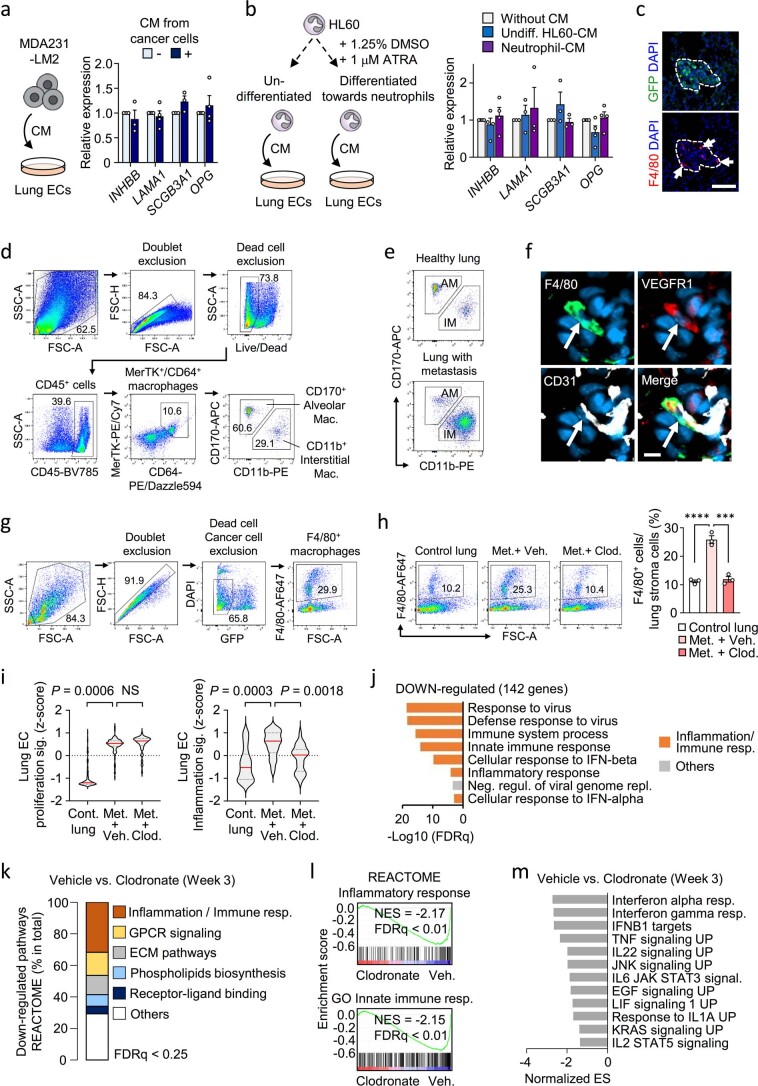
Functional role of macrophages in vascular niches. **a**, Expression of four vascular niche factors in human lung ECs treated with CM from MDA231-LM2 breast cancer cells. Schematic of the experiment (left) and relative expression (right). Values are means from 3 (*INHBB* and *SCGB3A1*) or 4 (*LAMA1* and *OPG*) independent experiments with s.e.m. **b**, Expression of the four vascular niche factors in human lung ECs treated with CM from HL60 cells; undifferentiated or differentiated towards neutrophils. Experimental setup (left) and relative expression as means with s.e.m. (right) from 3 (*LAMA1* and *SCGB3A1*) or 4 (*INHBB* and *OPG*) independent experiments are shown. **c**, Immunofluorescence analysis of macrophages (F4/80, arrows) in metastatic nodules in lungs, 2 weeks post intravenous injection of MDA231-LM2 cancer cells (GFP). Cell nuclei were stained by DAPI. Representative example from 4 independent experiments is shown. Scale bar, 100 μm. **d,e**, Flow cytometry analysis of alveolar (CD170^+^ CD11b^-^) and interstitial (CD170^-^ CD11b^+^) macrophage populations in control mouse lungs and lungs with 4T1-derived metastases. Example of gating strategy (d) and comparison between control and metastatic lungs (e) are shown. **f**, Immunofluorescence analysis of F4/80 and VEGFR1 expression (markers of interstitial macrophages when combined) and endothelial marker CD31 in lung metastasis. Representative example from 3 independent samples is shown. Scale bar, 10 μm. **g**,**h**, Flow cytometry analysis of F4/80^+^ macrophages in healthy control lung and metastatic lung (week 3) treated with PBS-liposome or clodronate-liposome. Gating strategy example (g) and quantification (h) are shown; *n*= 3 mice per group. *P* values were determined by one-way ANOVA with Dunnett’s multiple comparison test. ***P< 0.001 and ****P< 0.0001. **i**, Lung EC proliferation signature (Supplementary Table 3) and lung EC inflammation signature (Supplementary Table 4) expressed in ECs isolated from metastatic lungs under indicated conditions. *P* values were calculated with averaged z-score of genes within signatures by one-way ANOVA with Dunnett’s multiple comparison test; *n* = 3 for each group. NS, not significant. **j**, GO term analysis of down-regulated genes (log2FC< −0.75, FDR< 0.25) in lung ECs after macrophage-depletion. **k**, Composition of down-regulated REACTOME pathway gene clusters with FDR< 0.25. **l**, GSEA of “Inflammatory response” (C2 in MSigDB, top) and “Innate immune response” (C5 in MSigDB, bottom) signatures in lung ECs from mice treated with clodronate-liposome. NES, normalized enrichment score. **m**, Downregulated gene clusters of cellular signaling in lung ECs isolated from metastasis-bearing mice after macrophage depletion. GSEA was performed using Hallmark and C2 CGP in MSigDB. Signatures with FDR< 0.25 are shown. ES, enrichment score. Source data

To address whether breast cancer cells directly induce the vascular niche components, we treated lung ECs with CM from MDA231-LM2 cancer cells and analyzed the expression of niche genes. However, CM from cancer cells did not induce the niche factors in ECs (Extended Data Fig. 5a), indicating that another cell type is probably required for the induction. In light of this, we used GSEA to analyze the properties of lung metastasis samples stratified according to GSP58 (Fig. 6a) and observed that samples expressing high GSP58 showed enrichment of gene signatures of innate immune cells (Fig. 6b). Thus, we examined whether these cells might be inducers of the vascular niche and selected neutrophils and macrophages for further analysis. CM from an activated macrophage cell line (RAW264.7), but not a neutrophil cell line (HL60), promoted expression of niche components in lung ECs (Fig. 6c and Extended Data Fig. 5b). This suggested that macrophages could be intermediates for metastasis-induced changes in ECs. We performed immunofluorescence analysis of the F4/80 macrophage marker in metastatic nodules in lung, and observed substantial numbers of infiltrating macrophages frequently localized to blood vessels (Fig. 6d,e and Extended Data Fig. 5c). To analyze the identity of this macrophage population we used flow cytometry and determined the expression of CD170 and CD11b, which can distinguish between local alveolar macrophages (CD170^+^ CD11b^–^) and bone marrow-derived interstitial macrophages (CD170^–^ CD11b^+^). We analyzed macrophages sorted from healthy mouse lungs or from lungs harboring metastases by MDA231-LM2 (in NSG mice) or 4T1 (in BALB/c mice). These experiments showed that interstitial macrophages, but not alveolar macrophages, were increased during metastatic progression (Fig. 6f,g and Extended Data Fig. 5d,e). VEGFR1 expression, combined with F4/80, has also been demonstrated to mark interstitial macrophages^31^, and immunofluorescence analysis of VEGFR1, F4/80 and CD31 in metastatic nodules showed that VEGFR1 and F4/80 double-positive macrophages were found in proximity to CD31^+^ ECs (Extended Data Fig. 5f). This suggested that interstitial macrophages are probable inducers of the perivascular niche.

**Extended Data Fig. 6:**
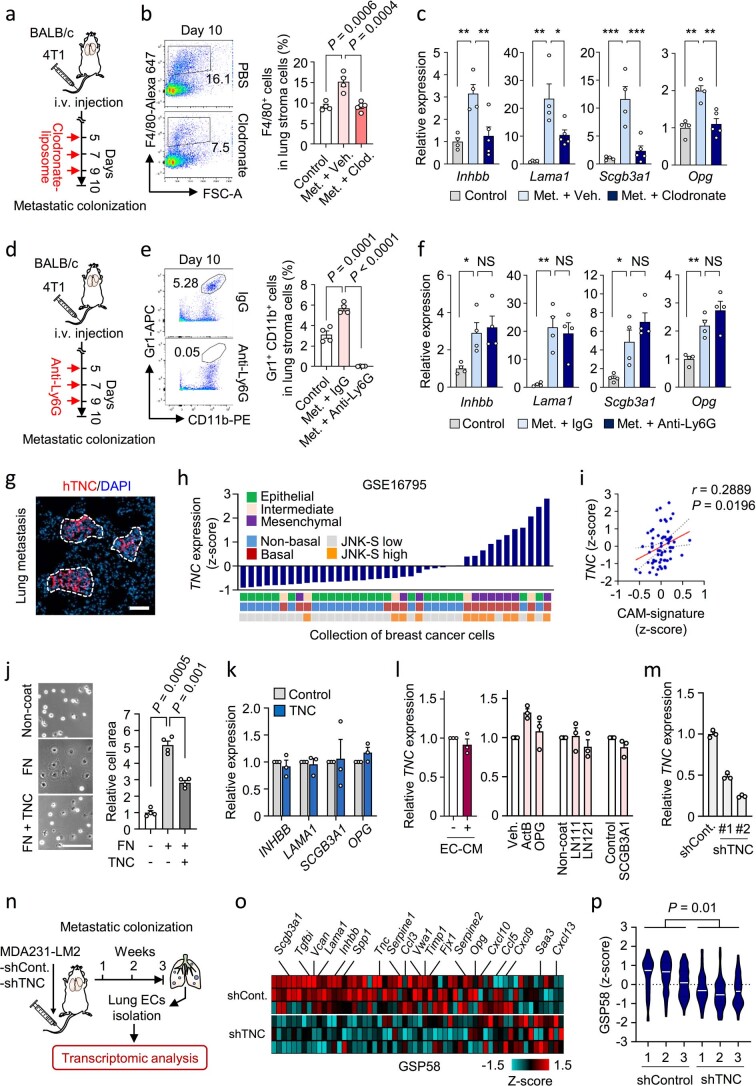
Analysis of innate immune cells and TNC in vascular niches in lungs. **a**, Scheme of macrophage-depletion by transient treatment with clodronate-liposome to BALB/c mice after intravenous injection of 4T1 cancer cells. **b**, Flow cytometry analysis of F4/80^+^ macrophages in metastatic lung treated with PBS-liposome or clodronate-liposome; *n* = 4 mice (control and metastasis with PBS-liposome (vehicle)) and *n* = 5 mice (metastasis with clodronate-liposome). Means with s.e.m. are shown. *P* values were determined by one-way ANOVA with Dunnett’s multiple comparison test. **c**, Expression of vascular niche components in lung ECs isolated from control healthy lung or metastatic lung treated with PBS-liposome or clodronate-liposome; *n* = 4 mice (control and metastasis with vehicle) and *n* = 5 mice (metastasis with clodronate-liposome). Shown are means with s.e.m. *P* values were calculated by one-way ANOVA with Holm–Sidak’s multiple comparison test. *P < 0.05, **P < 0.01 and ***P < 0.001. **d**, Scheme of neutrophil depletion by transient treatment with anti-Ly6G antibody in BALB/c mice after intravenous injection of 4T1 cells. **e**, Flow cytometry analysis of Gr1^+^CD11b^+^ neutrophils in metastatic lung treated with IgG or anti-Ly6G antibody; *n* = 4 mice per group. Data are means with s.e.m. *P* values were determined by one-way ANOVA with Dunnett’s multiple comparison test. **f**, Expression of niche factors in lung ECs isolated from control healthy lung or metastatic lung treated with IgG or anti-Ly6G antibody; *n*= 4 mice. Means with s.e.m. are shown. *P* values were calculated by one-way ANOVA with Holm-Sidak’s multiple comparison test. *P< 0.05 and **P< 0.01. NS, not significant. **g**, Immunofluorescence analysis showing accumulation of cancer cell-derived hTNC (human TNC) in metastatic nodule at 2 weeks after intravenous injection of MDA231-LM2 cells. Nuclei were stained by DAPI. Shown is a representative from 3 independent experiments. Scale bar, 100 μm. Dashed lines indicate nodule margins. **h**, TNC expression in breast cancer cell lines of different phenotype (epithelial/intermediate/mesenchymal), breast cancer subtype (Non-basal/Basal) and activity of JNK signaling. **i**, Correlation analysis of TNC and classically activated macrophage-signature (CAM-S) in 65 metastases samples from breast cancer patients. Linear regression with Pearson correlation r and two-tailed *P* values are shown. **j**, Analysis of EC spreading on fibronectin (FN) or TNC and FN coated plates. Scale bar, 200 μm. Shown are means with s.e.m. *P* values were determined with repeated measures one-way ANOVA with Dunnett’s multiple comparison test from 4 independent experiments. **k**, Expression of niche factors in lung ECs in response to TNC. Data are means with s.e.m. from 3 independent experiments. **l**, Expression of TNC in breast cancer cells stimulated with EC-derived CM or with indicated niche components. Shown are means with s.e.m. from 3 independent experiments. **m**, TNC expression in MDA231-LM2 cancer cells transduced with shControl or shTNC. Means with s.e.m. of 3 technical replicates from qPCR results are shown. **n-p**, GSP58 expression in ECs isolated from metastasis formed by shControl or shTNC MDA231-LM2 breast cancer cells. Experimental procedures (n), heatmap of GSP58 expression (o) and violin plot of GSP58 expression (p) in ECs from TNC knockdown metastases are shown. *P* values were determined by unpaired two-tailed t-test from 3 biological replicates. Source data

To investigate the functional role of macrophages in endothelial activation and metastatic colonization, we depleted macrophages from mice harboring growing metastases using clodronate-liposome (Fig. 6h and Extended Data Fig. 5g,h). We isolated lung ECs from the mice, performed transcriptomic analysis and observed that macrophage depletion repressed the expression of numerous genes within GSP58 in ECs, including *Inhbb*, *Lama1*, *Scgb3a1* and *Opg* (Fig. 6i–k). Interestingly, whereas expression of EC proliferation genes was unaffected by macrophage depletion, EC inflammatory responses were markedly repressed (Extended Data Fig. 5i–m). These results suggest that metastasis-associated macrophages induce inflammatory responses in lung ECs. In line with the findings from xenograft mouse models, elimination of macrophages in the fully immunocompetent 4T1 mouse mammary tumor model also repressed expression of the four niche genes, indicating that macrophages are a crucial regulator of vascular niche factors in the context of an intact immune system (Extended Data Fig. 6a–c). Furthermore, consistent with the in vitro results, no change in niche factor expression was observed following elimination of neutrophils in the 4T1 model (Extended Data Fig. 6d–f). Finally, depletion of macrophages significantly repressed metastatic colonization of the lung by MDA231-LM2 cancer cells (Fig. 6l). Together, these results indicate that perivascular macrophages function as regulators of the vascular niche during lung metastasis.

### TNC–TLR4-activated macrophages induce the endothelial niche

**Fig. 7:**
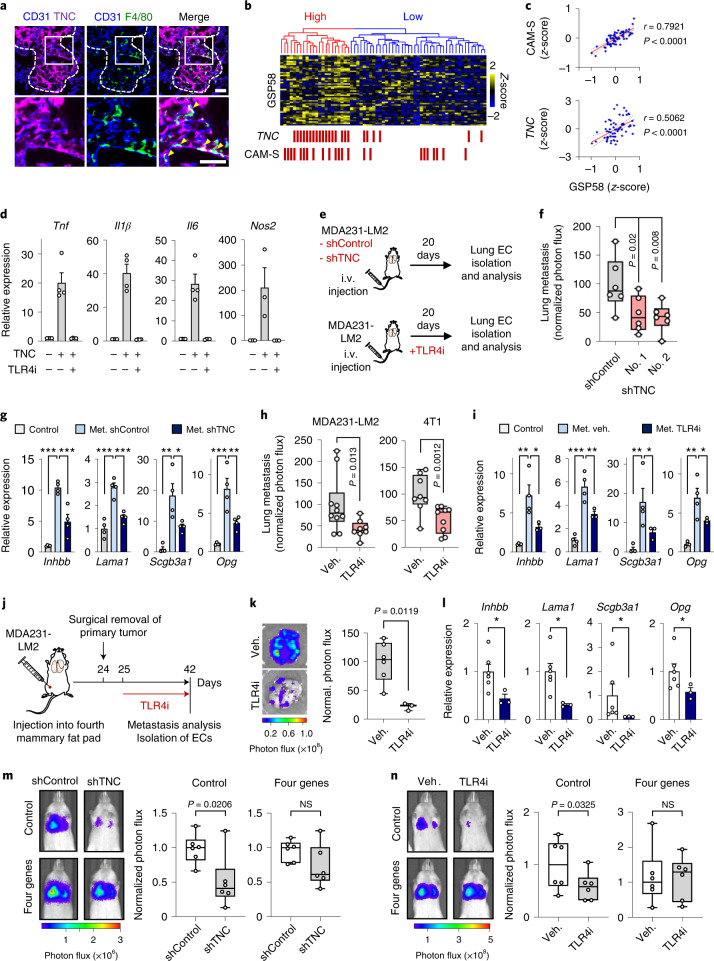
TNC–TLR4 axis promotes activation of perivascular macrophages and subsequent formation of a pro-metastatic endothelial niche in lung. **a**, Immunofluorescence analysis of TNC (purple), macrophages (F4/80, green) and ECs (CD31, blue) in MDA231-LM2 metastasis from mouse lung. Arrowheads indicate colocalization of TNC and macrophages at perivasculature. Dashed line indicates margins of metastasis. Shown are representative images from four independent samples. Scale bars, 50 μm. **b**, Hierarchical clustering of 65 human metastases of breast cancer (GSE14020) according to expression of GSP58. TNC- or classically activated macrophage-signature (CAM-S)-positive metastases are indicated by red bars. **c**, Correlation analysis of indicated parameters in 65 metastasis samples from patients with breast cancer. Linear regression with Pearson’s *r* and two-tailed *P* values are shown. **d**, Expression of indicated markers in macrophages treated with recombinant TNC or a combination of TNC and TLR4i (TAK-242) for 6 h; *n* = 3 (*Nos2*) or *n* = 4 (*Tnf*, *Il1b* and *Il6*) independent experiments. Shown are means with s.e.m. **e**, Experimental outlines showing MDA231-LM2 cancer cells, transduced with shRNA control (shControl) or shRNA against TNC (shTNC), injected intravenously (i.v.) into mice (top) or MDA231-LM2 cells injected i.v. into mice followed by treatment with TLR4i (bottom). **f**, Lung metastasis based on bioluminescence in mice injected with shControl or shTNC (two independent hairpins) transduced breast cancer cells; *n* = 6 mice for each group. *P* values calculated by one-tailed Mann–Whitney test. **g**, Expression of perivascular niche factors in ECs isolated from metastatic lung as in **f**; *n* = 4 mice. Data are means with s.e.m., and *P* values were determined by one-way ANOVA with Holm–Sidak’s multiple comparison test. *P < 0.05, **P < 0.01, ***P < 0.001. **h**, Bioluminescence analysis of mouse lungs harboring metastases and treated with TLR4i. For MDA231-LM2 and control, *n* = 10 mice; TLR4i, *n* = 8 mice; 4T1 and control, *n* = 8 mice; TLR4i, *n* = 9 mice. *P* values were calculated by one-tailed Mann–Whitney test. **i**, Expression of indicated genes in ECs isolated from lungs harboring MDA231-LM2 metastases as in **h**; *n* = 4 mice (control and metastasis with vehicle treatment) and *n* = 3 mice (metastasis with TLR4i treatment). Data are means with s.e.m., and *P* values were determined by one-way ANOVA with Holm–Sidak’s multiple comparison test. *P < 0.05, **P < 0.01, ***P < 0.001. **j**, Experimental outline of spontaneous lung metastasis in an orthotopic model. MDA231-LM2 cancer cells were implanted to the fourth mammary fat pad and their growth followed for 24 days, then tumors were removed surgically. Mice were treated with TLR4i from day 25 onwards. **k**, Metastasis in lungs of mice from **j**. Left, ex vivo bioluminescence; right, quantification of metastasis; *n* = 6 (vehicle) and *n* = 3 (TLR4i). *P* value was determined by one-tailed Mann–Whitney test. **l**, Expression of four niche factors in EC lungs isolated from mice harboring metastasis and treated with vehicle or TLR4i as in **j**,**k**. Data are means with s.e.m. obtained from *n* = 6 mice (vehicle) and *n* = 3 mice (TLR4i). *P* value was determined by one-tailed Mann–Whitney test. *P < 0.05. **d**,**g**,**i**,**l**, Expression was determined by qPCR. **m**,**n**, Rescue experiments where niche factors were ectopically expressed in metastatic breast cancer cells in the context of TNC knockdown (**m**) or TLR4i treatment (**n**) during lung colonization; *n* = 6 mice for each group. *P* values were determined by one-tailed Mann–Whitney test. **f**,**h**,**k**,**m**,**n**, Boxes show median with upper and lower quartiles, and whiskers indicate maximum and minimum values. Source data

**Extended Data Fig. 7:**
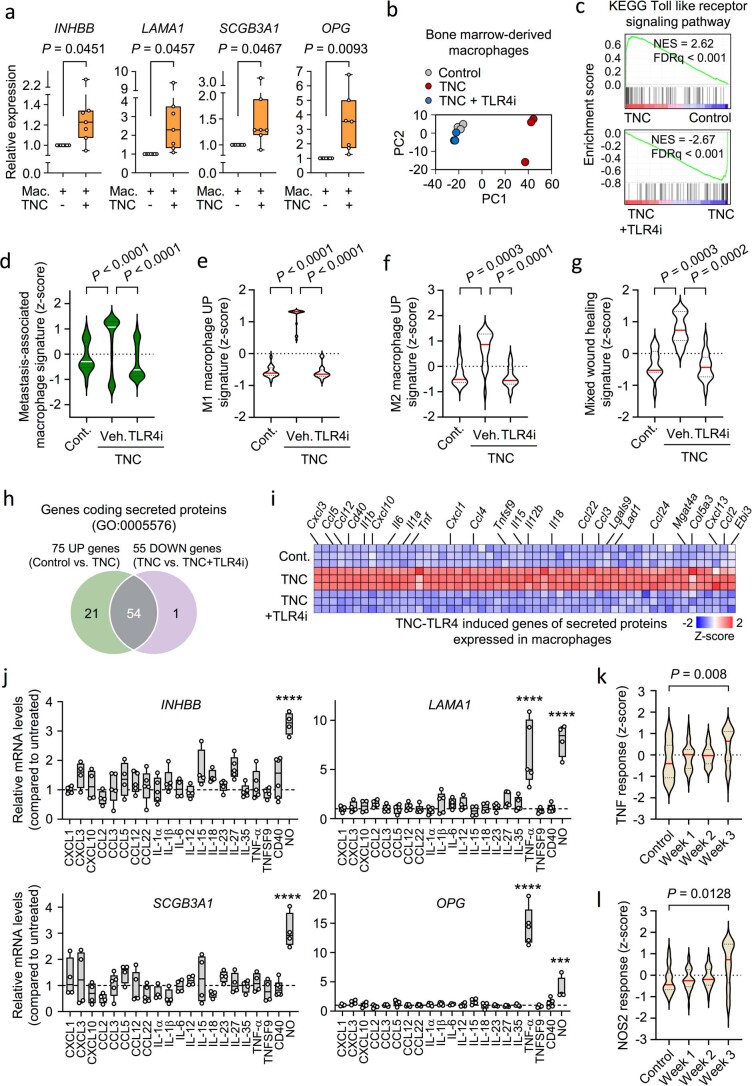
Macrophage phenotype and secreted factors in the vascular niche. **a**, Expression of *INHBB*, *LAMA1*, *SCGB3A1*, and *OPG* in ECs cocultured with control or TNC-stimulated macrophages. Expression was determined by qPCR. *P* values were calculated by paired one-tailed t test from 7 independent experiments. Boxes show median with upper and lower quartiles and whiskers indicate maximum and minimum values. **b**, Principal component analysis of bone marrow-derived macrophages treated with TNC or combination of TNC and TLR4i. **c**, GSEA showing enrichment of Toll-like receptor signaling pathway (KEGG) in macrophages treated with TNC or TNC+TLR4i. FDRs were determined from *P* values calculated by random gene set permutation test. **d**-**g**, Violin plot analyses of indicated gene signatures within the dataset of TNC-TLR4 stimulated macrophages. Gene signatures: metastasis-associated macrophages^39^ (up-regulated genes in metastasis-associated macrophages with log2FC> 2), M1 macrophages^40^, M2 macrophages^40^ and wound healing^41^. *P* values were determined by one-way ANOVA with Dunnett’s multiple comparison test from 3 biological replicates. **h**, Venn diagram showing number of genes of secreted proteins in bone marrow-derived macrophages, that are induced by TNC in a TLR4-dependent manner. **i**, Heatmap of genes induced by TNC and repressed by TLR4 inhibition. Selected examples are highlighted. **j**, Screen of 21 candidate factors to identify inducers of the four niche components. 20 candidates are from the list in panel i and one (NO, induced by diethylentriamine NONOate) was selected based on *Nos2* upregulation by TNC (Fig. 7d). ST1.6R endothelial cells were stimulated with indicated factors and expression of *INHBB*, *LAMA1*, *SCGB3A1* and *OPG* analyzed by qPCR. ***P< 0.001, ****P< 0.0001*. P* values were determined by one-way ANOVA with Dunnett’s multiple comparison test from minimum 4 independent experiments. Boxes show median with upper and lower quartiles and whiskers indicate maximum and minimum values. **k**,**l**, Violin plots analyzing expression of TNF response signature (SANA_TNF signaling, C2 in MSigDB) and NOS2 response signature (ZAMORA_NOS2 targets up, C2 in MSigDB) in ECs isolated from healthy mouse lungs or lungs harboring metastases. *P* values were calculated with an unpaired two-tailed t test from 3 biological replicates. Source data

The extracellular matrix is increasingly recognized as an important regulator of cancer progression and metastasis^32–34^. TNC is a matrix glycoprotein expressed by breast cancer cells as a crucial niche component and promoter of lung metastasis^35^. In breast cancer cells, TNC expression is linked to both the basal subtype and a mesenchymal phenotype (Extended Data Fig. 6g,h and Supplementary Table 9). We used immunofluorescence analysis to explore the localization of TNC with respect to metastasis-associated macrophages and ECs, and observed that TNC colocalizes with both cell types in metastatic nodules (Fig. 7a). Moreover, in metastases from patients with breast cancer, TNC expression—or expression of a gene signature from activated macrophages^36^—overlapped GSP58 expression and with each other in patient samples and the expression levels correlated (Fig. 7b,c and Extended Data Fig. 6i). This suggested a link between TNC, metastasis-associated macrophages and activation of vascular niches in human metastases. Interestingly, previous studies have shown that TNC can bind and activate TLR4 in different cell types, leading to inflammatory signaling in mouse models of arthritis^37^. In line with these findings, we observed that macrophages treated with recombinant TNC induced expression of *Tnf*, *Il1b, Il6* and *Nos2* as activation markers, and this was reversed by TLR4 inhibitor (TLR4i) (Fig. 7d). However, although recombinant TNC can affect the adhesive properties of ECs, it did not directly induce expression of vascular niche components (Extended Data Fig. 6j,k). Furthermore, TNC expression in breast cancer cells was unaffected by treatment with EC-CM or recombinant vascular niche factors (Extended Data Fig. 6l). Considering these results in the context of vascular niche-regulation by macrophages, we hypothesized that perivascular TNC might be involved in the activation of macrophages to induce vascular niche components in metastasis. To address this in vivo, we injected control or TNC knockdown MDA231-LM2 cancer cells intravenously into NSG mice (Fig. 7e, top and Extended Data Fig. 6m). Consistent with previous studies^35,38^, TNC deficiency repressed metastatic colonization by breast cancer cells (Fig. 7f). We isolated ECs from mice harboring control or TNC knockdown metastases using FACS and determined the expression of GSP58 by microarrays (Extended Data Fig. 6n). The majority of GSP58 genes was repressed in TNC knockdown metastases (Extended Data Fig. 6o,p). Furthermore, quantitative PCR (qPCR) analysis of ECs isolated from mice confirmed that expression of the four niche factors was induced in metastases in a TNC-dependent manner (Fig. 7g). To further investigate direct TNC effects on macrophages and subsequent activation of endothelial niches, we cocultured ECs with TNC-treated RAW264.7 macrophages and analyzed expression of the four niche components. TNC-treated macrophages induced the niche factors in cocultured ECs (Extended Data Fig. 7a). In light of this, we aimed to uncover which factors from TNC-activated macrophages were responsible for induction of the vascular niche. We stimulated macrophages with TNC, alone or in combination with TLR4i, and performed transcriptomic analysis. PCA revealed major TNC-induced changes that were reversed by TLR4i cotreatment (Extended Data Fig. 7b). GSEA showed that a Toll-like receptor signature (KEGG) was enriched in TNC-stimulated macrophages and was under-represented when TLR4i was included (Extended Data Fig. 7c). Moreover, violin plot analysis showed induction of a metastasis-associated macrophage signature^39^ by TNC in a TLR4-dependent manner (Extended Data Fig. 7d). Further investigation of gene signatures associated with macrophage phenotypes^40,41^ showed that TNC can induce changes linked to different phenotypes such as the classical inflammatory macrophage phenotype (M1), alternatively activated macrophages (M2) and a wound-healing phenotype (Extended Data Fig. 7e–g). To address potential paracrine effects of TNC-stimulated macrophages, we analyzed genes of secreted proteins and observed induction of 75 by TNC, of which 54 were dependent on TLR4 (Extended Data Fig. 7h,i and Supplementary Table 10). To address whether TNC-induced factors from macrophages could promote niche components in ECs, we investigated a set of 20 of these cytokines and, since *Nos2* was induced by TNC in a TLR4-dependend manner (Fig. 7d), we also included a NO-inducing NONOate as a stimulant. Of these factors, only NONOate upregulated *INHBB* and *SCGB3A1* whereas both NONOate and TNF-α induced *LAMA1* and *OPG* (Extended Data Fig. 7j). Importantly, ECs from metastatic nodules in mice showed indications of high responses to both TNF and NO (Extended Data Fig. 7k,l). These results suggest that NO and TNF produced by macrophages can activate the vascular niche.

**Extended Data Fig. 8:**
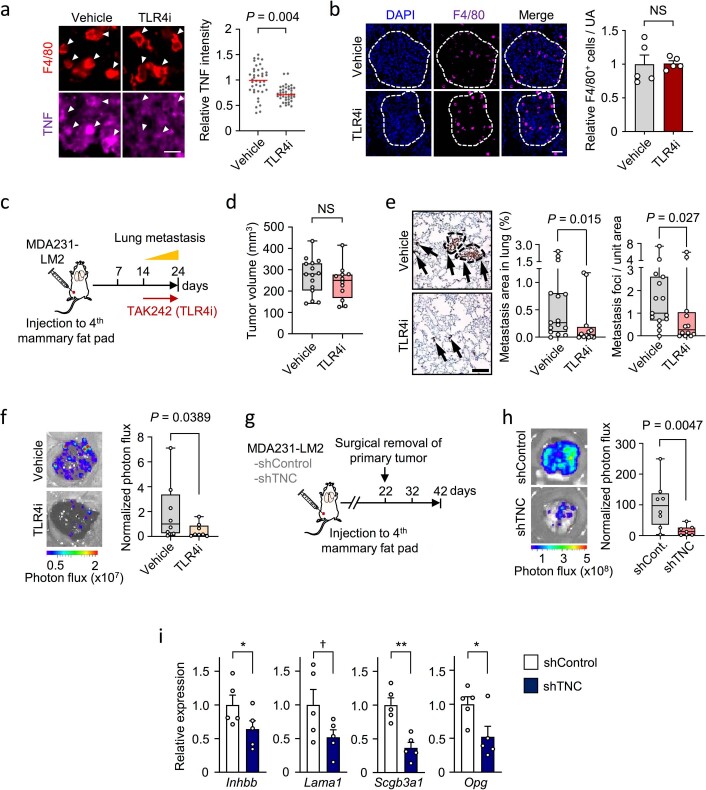
Analysis of TNC-TLR4 axis in lung metastasis. **a**, Immunofluorescence analysis of TNF in F4/80^+^ macrophages within MDA231-LM2 metastatic nodules from mice treated with vehicle or TLR4i. Representative examples (left) and quantification of relative signals (right) from 5 mice. *P* value was determined by one-tailed Mann-Whitney test. Scale bar, 20 μm. **b**, Immunofluorescence analysis of macrophages (F4/80) in MDA231-LM2 lung metastases from mice treated with vehicle or TLR4i. Dashed lines indicate metastases margins. Relative number of F4/80^+^ cells per unit area (UA) of metastatic foci was quantified, and means with s.e.m. are shown (right). Statistical analysis was performed with one-tailed Mann-Whitney test; *n*= 5 mice. Scale bar, 50 μm. **c**, Experimental outline of spontaneous lung metastasis assay in an orthotopic model where tumor-bearing mice were treated with TLR4i. **d**, Primary tumor volume measured on day 24, Vehicle: *n*= 15 mice, TLR4i: *n*= 12 mice. Statistical analysis was performed with one-tailed Mann-Whitney test. **e**, Spontaneous metastases in lungs of mice on day 24 as in c. Left, representative examples of metastases (arrows) marked by expression of human vimentin in cancer cells. Right, quantification of lung metastasis area and nodule numbers based on human vimentin marker expression; Vehicle: *n*= 15 mice, TLR4i: *n*= 12 mice. *P* values were calculated by one-tailed Mann-Whitney test. Scale bar, 200 μm. **f**, Liver metastasis in an orthotopic metastasis model where breast cancer cells were implanted into mammary fat pads and mice treated with TLR4i; *n*= 8 mice for each group. *P* value was calculated by one-tailed Mann-Whitney test. **g**,**h**, Analysis of lung metastasis in TNC knockdown orthotopic metastasis model where primary tumor was removed surgically at day 22. Diagram of experimental procedures (g). Quantification of lung metastasis based on bioluminescence in mouse lungs (h), shControl: *n*= 8 mice, shTNC: *n*= 7 mice. *P* value was determined by one-tailed Mann-Whitney test. **i**, Expression of indicated niche components in ECs isolated from mice as in panels g,h; *n*= 5 for each group. Shown are means with s.e.m., and *P* values were calculated by one-tailed Mann-Whitney test. *P< 0.05, **P< 0.01 and ^†^*P*= 0.075. Boxes in panels d-f and h show median with upper and lower quartiles and whiskers indicate maximum and minimum values. Source data

To address the functional role of TLR4 as a regulator of vascular niches, we treated metastasis-bearing mice with TLR4i and isolated ECs for analysis (Fig. 7e, bottom). Whereas treatment with TLR4i did not affect recruitment of macrophages, it significantly reduced activation (Extended Data Fig. 8a,b). Importantly, inhibition of TLR4 caused downregulation of the vascular niche components and impeded metastatic colonization of the lungs (Fig. 7h, left and 7i). Considering the crosstalk between innate and adaptive immunity, we addressed the functional role of TLR4 in the immunocompetent 4T1 mammary tumor model. In line with results from xenograft models, TLR4i treatment inhibited metastatic colonization in the 4T1 model (Fig. 7h, right), and these results were crucially reproduced in an orthotopic mouse model of spontaneous metastasis to the lung (Extended Data Fig. 8c). Whereas mammary tumor growth was not significantly affected by TLR4 inhibition, both the size and number of metastatic nodules were reduced by TLR4i (Extended Data Fig. 8d,e). Notably, analysis of livers in the orthotopic mouse model showed significantly reduced liver metastasis following TLR4i treatment (Extended Data Fig. 8f). To provide evidence from a model that would mimic clinical settings, we analyzed metastatic progression and expression of the four niche components in ECs from mice where primary tumors had been surgically removed before analysis of metastasis. The experiments were done in the context of either TNC knockdown or TLR4i-treated metastases, after primary tumor removal (Fig. 7j and Extended Data Fig. 8g). Both TNC knockdown and TLR4i treatment reduced metastatic burden in the lung and repressed expression of the four niche components in lung ECs (Fig. 7k,l and Extended Data Fig. 8h,i). Finally, we investigated whether overexpression of the four niche factors could circumvent macrophage activation and restore diminished metastases in the absence of TNC or TLR4 function. Indeed, ectopic expression of the four niche components rescued both TNC knockdown and TLR4 inhibition in metastasis-bearing mice (Fig. 7m,n). Together, these results suggest a stromal interaction in metastasis where cancer cells produce TNC that activates macrophages which consequently promote the formation of a pro-metastatic vascular niche.

### Combined inhibition of TLR4 and VEGF impedes lung metastasis

**Fig. 8:**
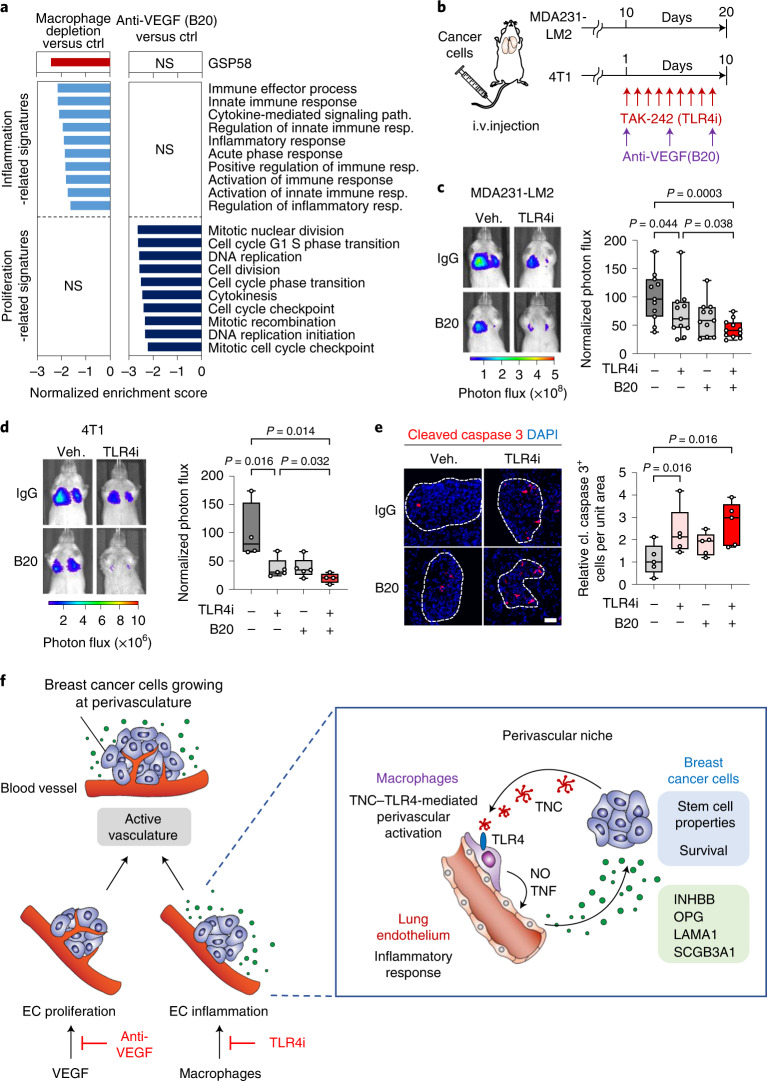
TLR4 inhibition and anti-VEGF therapy target different vascular functions, with combination treatment further impeding lung metastasis. **a**, GSEA of GSP58, inflammation- or proliferation-related signatures expressed in ECs from mice with either macrophage-depleted lung metastases (clodronate-liposome) or metastases treated with anti-VEGF therapy (B20). **b**–**d**, Inhibition of TLR4 and VEGF in mice harboring lung metastasis. **b**, Experimental outline where mice were intravenously injected with indicated breast cancer cells and treated with TLR4i or B20 as single treatments, or together as a combination treatment. **c**,**d**, Bioluminescence analysis of metastatic colonization of lungs in mice injected with MDA231-LM2 (**c**) or 4T1 (**d**) cancer cells and treated as described in **b**. Left, representative bioluminescence images; right, quantification of metastatic lung colonization based on bioluminescence signal. MDA231-LM2, *n* = 11 mice; 4T1, *n* = 5 mice (single TLR4i or B20 treatments) and *n* = 4 mice (control or double treatment). *P* values were calculated by one-tailed Mann–Whitney test. **e**, Immunofluorescence analysis of apoptosis by expression of cleaved caspase 3 in metastatic nodules treated with TLR4i, B20 or a combination of the two; *n* = 5 mice for each group. Scale bar, 50 μm. *P* values were determined by one-tailed Mann–Whitney test. **c**–**e**, Boxes show median with upper and lower quartiles, and whiskers indicate maximum and minimum values. **f**, Model depicting two regulatory arms of vascular activation, where VEGF promotes proliferation of ECs and macrophages, stimulated by TNC–TLR4 signaling, promote inflammatory reaction in ECs and secretion of pro-metastatic factors of the vascular niche that are utilized by cancer cells. Shown are interactions between breast cancer cells and macrophages via the TNC–TLR4 axis, which lead to macrophage activation and subsequent induction of the perivascular niche by NO and TNF to produce pro-metastatic factors including INHBB, OPG, LAMA1 and SCGB3A1. INHBB and SCGB3A1 induce stem cell properties in breast cancer cells while OPG and LAMA1 promote survival of cancer cells at the metastatic site. Source data

**Extended Data Fig. 9:**
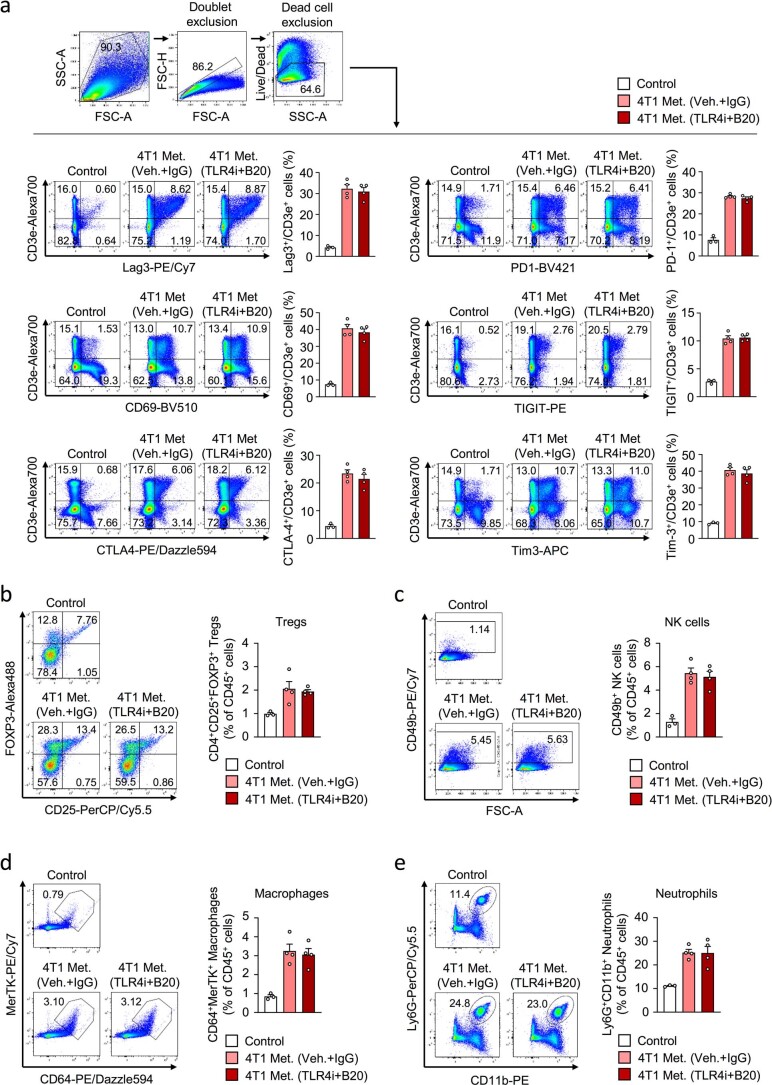
Immune response in metastatic lungs treated with inhibitors of TLR4 and VEGF. **a**, Flow cytometry analysis of T cell exhaustion in control lungs or lungs from 4T1 metastasis-bearing BALB/c mice after treatment with TLR4i and anti-VEGF (B20). Following exhaustion markers were analyzed on T cells: Lag3, CD69, CTLA-4, PD-1, TIGIT and Tim-3. Shown are flow cytometry examples and quantification of the indicated exhaustion markers expressed by T cells. Data are means with s.e.m. **b-e**, Flow cytometry analysis of regulatory T cells (Tregs, CD25^+^/FOXP3^+^), natural killer cells (NK cells, CD49b^+^), macrophages (CD64^+^/MerTK^+^) and neutrophils (Ly6G^+^/CD11b^+^) in control lungs or lungs with 4T1 metastasis in BALB/c mice upon treatment with TLR4i and B20. Shown are flow cytometry examples (left) and quantifications as means with s.e.m. (right). Analysis was performed on CD4^+^ gated cells (Tregs analysis) or CD45^+^ gated cells (analysis of NK cells, macrophages or neutrophils). Control group, *n*= 3 mice and metastasis groups treated with vehicle/IgG or TLR4i/B20, *n*= 4 mice each group. Source data

**Extended Data Fig. 10:**
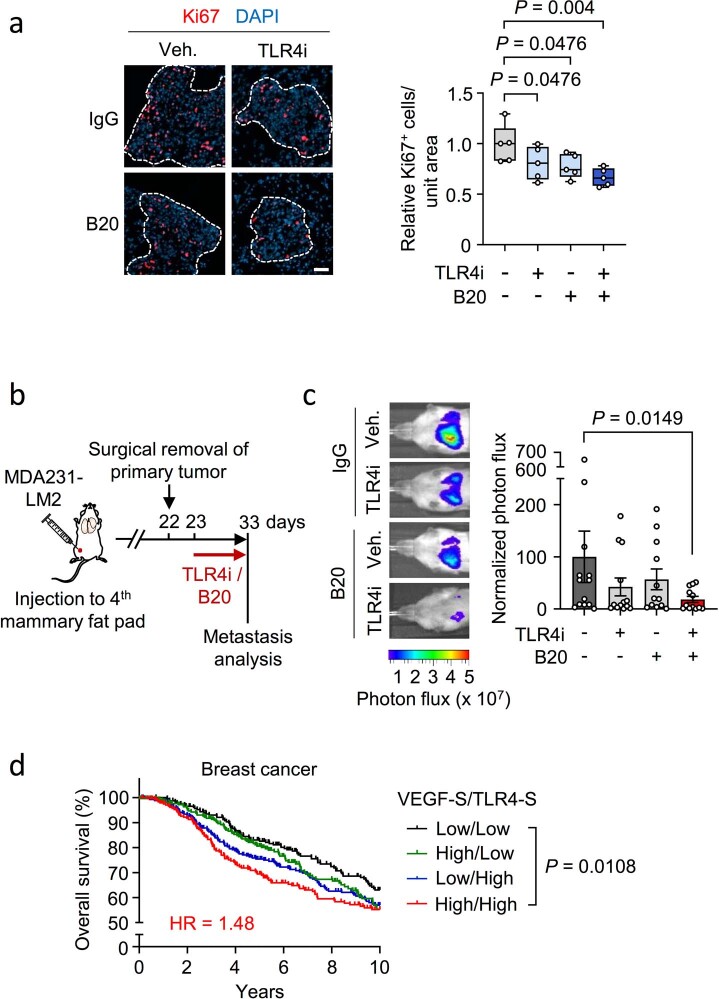
TLR4 and VEGF function in breast cancer metastasis. **a**, Immunofluorescence analysis of Ki67 expression in metastatic nodules after lung colonization by MDA231-LM2 breast cancer cells followed by treatment with TLR4i, anti-VEGF (B20) or combination of the two. Left, representative fluorescence images. Right, quantification; *n*= 5 mice for each group. Boxes show median with upper and lower quartiles and whiskers indicate maximum and minimum values. *P* values were determined by one-tailed Mann-Whitney test. Scale bar, 50 μm. **b**,**c**, Metastasis analysis in an orthotopic metastasis model where mice were treated with TLR4i, B20 or both, after the primary tumors had been surgically removed. Shown are experimental procedures (b) and quantification of metastasis (c) with representative examples of bioluminescence in lungs (left) and normalized photon flux (right); Control *n*= 13 mice; TLR4i *n*= 13 mice; B20 *n*= 12 mice; TLR4i + B20 *n*= 12 mice. Data are means +/- s.e.m. *P* value was calculated by one-tailed Mann-Whitney test. **d**, Kaplan Meier analysis showing overall survival of breast cancer patients (METABRIC discovery) stratified according to expression of VEGF signature (VEGF-S)^42^ and TLR4 signature (TLR4-S)^43^. Survival analysis spanning 10 years is shown. Patients were divided based on the median value of each signatures; Patient groups: VEGF-S/TLR4-S High/High *n*= 239; High/Low *n*= 259; Low/High *n*= 264; Low/Low *n*= 233. Shown is hazard ratio (HR) for the comparison VEGF-S/TLR4-S low/low vs high/high. *P* value was determined by Log-rank test. Source data

Our findings indicated a distinction between EC genes regulated by VEGF and those regulated by activated macrophages. To address this further, we analyzed transcriptomic changes in ECs isolated from metastatic lungs where mice had been depleted of macrophages, and compared these to expression changes in ECs from mice treated with anti-VEGF antibody. The results from GSEA show that macrophage-depended EC gene induction is particularly linked to inflammatory responses and GSP58, whereas VEGF-dependent induction leads to stimulation of proliferation (Fig. 8a). In light of this, we examined whether a combination of anti-TLR4 and anti-VEGF therapy could provide increased efficacy in suppression of metastasis. We treated xenograft and syngeneic metastasis-bearing mice with TLR4i and anti-VEGF antibody, either individually or together (Fig. 8b). The results showed that combined inhibition of TLR4 and VEGF provides improved efficacy in repression of metastasis compared to single treatments (Fig. 8c,d). Considering a potential effect of the combination treatment on tumor-induced immune responses, we analyzed T cell exhaustion markers and the presence of regulatory T cells (Tregs), natural killer cells (NK cells), macrophages and neutrophils—in a syngeneic mouse model of metastasis (the 4T1 model) where mice were treated with TLR4i and anti-VEGF antibody. Although we observed induction of all these markers in metastases, the combination treatment did not affect the responses (Extended Data Fig. 9a–e). However, we observed increased apoptosis and reduced proliferation in cancer cells of metastases following combination treatment (Fig. 8e and Extended Data Fig. 10a). We treated mice with TLR4i and anti-VEGF antibody in an orthotopic setting, where mammary fat pad tumors were surgically removed on day 22 post implantation and treatment was initiated on day 23 (Extended Data Fig. 10b). Combined TLR4i and anti-VEGF treatment significantly repressed lung metastasis (Extended Data Fig. 10c). Notably, analysis of the discovery dataset from the METABRIC study revealed that expression of VEGF and TLR4 signatures^42,43^ in patient samples is associated with poor overall survival (Extended Data Fig. 10d). Together, our results describe distinct endothelial activation properties where macrophage-mediated inflammation induces the production of vascular niche proteins and VEGF signaling promotes EC proliferation (Fig. 8f). These results provide a rationale to explore the combination of TLR4i with anti-VEGF therapy in suppression of vascular functions in metastases.

## Discussion

In this study we describe characteristic changes in lung ECs induced during metastatic colonization. We demonstrate a crucial role of specific molecular crosstalk at perivascular sites among colonizing breast cancer cells, macrophages and vascular endothelium during lung metastasis. The study provides insights into vascular regulation during metastasis, revealing a distinction between VEGF-induced proliferation and macrophage-induced inflammatory responses. Global transcriptomic analysis showed that VEGF-regulated gene expression patterns linked to cell proliferation and angiogenesis are induced in metastasis-associated lung ECs. However, the results also revealed an inflammatory reaction of ECs leading to production of numerous secreted proteins (GSP58) that are largely independent of VEGF, among which we identified INHBB, LAMA1, SCGB3A1 and OPG as crucial secreted components of the vascular niche in metastasis.

Growing evidence indicates that ECs play an important role in intercellular communication during development, tissue homeostasis and disease^6^. Transmembrane or secreted endothelial factors involved in cellular crosstalk have been termed angiocrine factors and can influence cancer progression^44^. For example, E-selectin produced by ECs in bone can promote epithelial-to-mesenchymal transition and stem cell properties in cancer cells to further metastatic colonization^45^. ECs can also produce specific extracellular matrix proteins that regulate states of dormancy and activation in disseminated breast cancer cells, and may enhance resistance to therapeutic intervention^9,46^. Antiangiogenic therapy targeting VEGF has been applied as part of a regimen to treat patients with advanced breast cancer. However, the clinical benefits from VEGF neutralization in patients with breast cancer have been limited^47,48^. Our results suggest that, although anti-VEGF therapy represses EC proliferation, it may not be able to block specific angiocrine functions of ECs during lung metastasis. However, the results also indicate that combined inhibition of VEGF-mediated proliferation and macrophage-induced vascular niche formation may be a promising approach to suppress broad endothelial functions in metastasis.

Our findings suggest that a crosstalk among disseminated breast cancer cells, activated macrophages and ECs is crucial to the formation of pro-metastatic vascular niches in the lung. Macrophages are activated by cancer cell-derived TNC and subsequently stimulate lung ECs by secreting NO and TNF to invoke vascular niche formation. TNC is recognized as a promoter of cancer progression in numerous malignancies and is expressed in highly aggressive cancer cells^49,50^. In metastatic breast cancer cells, TNC expression has been shown to be induced by JNK signaling and can be curbed by the metastasis-suppressing microRNA, miR335 (refs. ^38,51^). In addition to exerting changes in tumor stroma, TNC expressed by breast cancer cells functions in an autocrine manner, modulating Notch and Wnt signaling components to promote metastatic fitness of the cells^35^. Here, we reveal how TNC produced by cancer cells initiates activation of macrophages via TLR4 leading to induction of vascular niches. Notably, TNC-induced TLR4 activation has been shown to occur in the context of arthritis^37^. TNC is composed of multiple domains, among which the C-terminal fibrinogen globe was demonstrated to bind and activate TLR4 (refs. ^37,52^). In growing metastases, cancer cells are not the only source of TNC as activated fibroblasts can produce high TNC levels^50^. However, studies show that expression of TNC by reactive fibroblasts occurs at late time points and thus, in early metastasis, cancer cells are the primary source of TNC^35^. Although TNC from fibroblasts may contribute to vascular niches, our results indicate that TNC produced by cancer cells is crucial for the activation process.

Growing primary tumors can invoke specific changes in secondary organs, before colonization, leading to the generation of a premetastatic niche that facilitates colonization of these organs^53^. Some of these changes may affect blood vessels, as recently shown for leucine-rich alpha-2-glycoprotein 1 (ref. ^54^). However, our findings indicate that the four vascular niche genes are not induced by secreted factors from primary tumors. We conclude that TNC, produced by cancer cells invading the lungs, jump-starts the cascade that results in the formation of a pro-metastatic vascular niche.

Macrophages are recognized to express heterogeneous phenotypes depending on origin and function. Our results suggest that pro-metastatic macrophages that play a regulatory role for vascular niches in the lung are interstitial—that is, recruited from blood circulation—rather than local alveolar macrophages. This is in line with previous findings suggesting that alveolar macrophages are dispensable for metastatic progression whereas those recruited from the blood circulation are required for metastasis^31^. We explored the macrophage phenotype induced by TNC and observed induction of the classical inflammatory phenotype (M1), as well as features of alternatively activated macrophages (M2) and wound-healing properties. It is important to note the growing evidence which suggests that polarization of macrophages may be more complex and plastic than previously understood^55^. For example, single-cell analysis within the breast tumor microenvironment has shown that tumor-associated macrophages can exhibit both M1 and M2 features simultaneously^56^.

In conclusion, our findings delineate a multifaceted cellular crosstalk within growing metastases in the lung. The results show a crucial role for the vascular niche during metastatic progression, and emphasize the role of the extracellular matrix in its regulation. These interactions in metastatic nodules may serve as useful targets when developing future therapies against metastatic disease.

## Methods

### Human metastasis samples

Studies on breast cancer metastases were approved by the ethics committee of the University of Heidelberg Medical Faculty (no. S-716/2018) and conformed to the principles of the WMA Declaration of Helsinki and the Department of Health and Human Services Belmont Report. Patients gave written informed consent.

### Cell culture

Cell lines MDA-MB-231 (MDA231, American Type Culture Collection (ATCC)), MDA-MB-231-LM2 (MDA231-LM2, RRID:CVCL_5998, provided by J. Massagué)^10^ and 4T1, HEK293T and RAW264.7 (all from ATCC) were cultured in DMEM (GlutaMAX, Thermo Fisher Scientific) with 10% fetal bovine serum (FBS, Gibco). SUM159 (Asterand Bioscience) and SUM159-LM1 cells^51^ were maintained in DMEM/F12 medium (Thermo Fisher Scientific) with 5% FBS and 5 μg ml^–1^ human insulin (Sigma-Aldrich). E0771 (CH3 BioSystems) and HL60 (ATCC) cells were cultured in RPMI 1640 medium (Thermo Fisher Scientific) with 10% FBS; E0771 medium also included 10 mM HEPES (Sigma-Aldrich). Primary human pulmonary ECs (Lonza) were cultured in EBM-2 medium with the EBM-2 bullet kit (Lonza). The human pulmonary EC line ST1.6R^57^ was provided by R. E. Unger and C. J. Kirkpatrick, and cultured on collagen type I-coated plates with M199 (Thermo Fisher Scientific) containing 20% FBS, 25 μg ml^–1^ EC growth supplement (Corning) and 25 μg ml^–1^ heparin (Sigma-Aldrich). Bone marrow-derived macrophages were isolated from 8–10-week-old BALB/c mice and cultured in DMEM with 10% FBS and 20 ng ml^–1^ macrophage colony-stimulating-factor (M-CSF, Peprotech). All media contained 50 U ml^–1^ penicillin and 50 μg ml^–1^ streptomycin (Sigma-Aldrich). All cancer cell lines were authenticated using Multiplex Cell Authentication by Multiplexion Heidelberg.

### Mouse metastasis models

Female nonobese, diabetic, severe combined immunodeficiency gamma^null^ (NSG, The Jackson Laboratory), BALB/c (Janvier Labs or Envigo) or C57BL/6 mice (6–12 weeks old) were used for in vivo studies. Mice were housed in individually ventilated cages under controlled temperature (22 °C) and humidity (50%) under a 12/12-h light/dark cycle. All experiments with mice were conducted according to German legal regulations, and protocols were approved by the governmental review board of the state of Baden-Wuerttemberg, Regierungspraesidium Karlsruhe, under authorization nos. G-51/13, G-81/16 and G-98/18.

To track cancer cells in mice, cell lines MDA231, MDA231-LM2, SUM159 and SUM159-LM1, and mouse mammary tumor cell line E0771, were transduced with a triple-reporter-expressing herpes simplex virus thymidine kinase 1, GFP and firefly luciferase genes^58^. 4T1 was transfected with the nS/MARt-Luc vector, which was generated by exchanging the reporter gene GFP for luciferase in the SP-nS/MARt vector^59^.

For lung colonization assays, either 200,000 cells (MDA231-LM2 or SUM159-LM1), 25,000 cells (MDA231), 100,000 cells (SUM159 or 4T1) or 500,000 cells (E0771) were suspended in 100 μl of PBS and injected intravenously. Metastatic colonization was monitored by bioluminescence from luciferase. Mice were injected intraperitoneally with d-luciferin (150 mg kg^–1^, Biosynth) and imaged with an IVIS Spectrum (Caliper Life Sciences). Bioluminescence was analyzed using Living Image software v.4.4 (Caliper Life Sciences).

For the spontaneous metastasis assay in an orthotopic mouse model, 500,000 MDA231-LM2 cells were suspended in a 1:1 (v/v) mixture of Growth Factors-Reduced Matrigel (Corning) and PBS and implanted bilaterally into the fourth mammary fat pad. Primary tumor growth was monitored using a digital caliper and/or bioluminescence imaging. Tumor volume was calculated with the following formula: volume = length × width^2^ × 0.52. The maximal tumor volume permitted, 520 mm^3^, was not exceeded. Primary tumors were surgically removed at 22–24 days post implantation and spontaneous metastasis monitored until days 33–42. In experiments where primary tumors were not removed, mice were sacrificed after 24 days and metastatic burden analyzed.

### Drug treatment in vivo

To inhibit VEGF signaling in vivo, mice harboring lung metastases were injected intraperitoneally with anti-human/mouse VEGF-A neutralizing antibody (Genentech, no. B20.4.1.1; 5 or 10 mg kg^–1^)^15^ twice per week for 3 weeks, or with anti-mouse VEGFR2-blocking antibody (DC101, BioXcell) at 40 mg kg^–1^, on days 21 and 24 post cancer cell implantation. To deplete macrophages, mice were treated with clodronate-liposome (Liposoma BV) by intravenous injection of 150 μl every second day. PBS-loaded liposome (Liposoma BV) was used as control. Neutrophils were depleted by anti-Ly6G antibody (1A8, BioXcell) injected intraperitoneally (20 mg kg^–1^) every second day. For TLR4 inhibition, TAK-242 (Merck Millipore) was injected intraperitoneally at 10 mg kg^–1^ once per day. Combined TLR4 and VEGF inhibition was performed by intraperitoneal injection of TAK-242 (10 mg kg^–1^, once daily) and B20.4.1.1 (10 mg kg^–1^, twice weekly).

### Isolation of ECs from mouse organs

Mouse organs were digested using 0.5% Collagenase type III (Pan Biotech), 1% Dispase II (Gibco) and 30 μg ml^–1^ DNase I in PBS for 45 min at 37 °C. Cells were resuspended in PBS containing 2% FBS and 2 mM EDTA, and sequentially filtered through 100- and 70-μm nylon filters (Greiner). ACK Lysing Buffer (Lonza) was used to remove red blood cells. Cells were resuspended in PBS containing 2% FBS and 2 mM EDTA for FACS, or DMEM containing 0.1% BSA for magnetic-activated cell sorting (MACS).

For FACS, cells were incubated (30 min on ice) with the following antibodies: phycoerythrin (PE)-conjugated anti-CD45 (1:3,000), PE-conjugated anti-CD326 (1:250), allophycocyanin (APC)-conjugated anti-CD140a (1:50), APC-conjugated anti-CD140b (1:50) and PE/Cyanine7 (Cy7)-conjugated anti-CD31 (1:500) (all from eBioscience) and PE-conjugated anti-CD11b (1:3,000, BD Biosciences). Cell sorting was performed with either a BD FACSAria I or FACSAria II machine (Becton Dickinson) with BD FACSDiva software 8.0.1. GFP^–^ (cancer cells), PE^–^, APC^–^ and PE/Cy^+^ population was isolated as the lung EC fraction. Fibroblasts (CD140a^+^ and b^+^), bone marrow-derived cells (CD45^+^) and epithelial cells (CD326^+^) were also isolated, and expression of vascular niche factor candidates analyzed by qPCR. For MACS-based isolation of ECs, rat anti-mouse CD31 antibody (Mec13.3, BD Pharmingen) was coupled to sheep anti-rat IgG dynabeads (Thermo Fisher Scientific) and used to purify ECs.

### Oncosphere treatment and analysis

Oncosphere cultures were stimulated with CM from ECs, either with CM from HEK293T containing secreted niche factors or with recombinant niche factors. EC-CM: CM was collected from ST1.6R ECs (cultured in HuMEC medium (Invitrogen) with 0.1% BSA for 24 h). For oncosphere formation assays with CM containing secreted vascular niche factors, HEK293T cells were used to generate CM. HEK293T-CM: HEK293T cells were transfected with pLVX-Puro vector (Invitrogen) expressing human INHBB, SCGB3A1 or OPG using Lipofectamine 2000 (Invitrogen), according to the manufacturer’s instructions. All collected CM was filtered with a 0.45-μm syringe-filter (Techno Plastic Products). To prepare CM containing recombinant LAMA1, HEK293T cells were infected with lentivirus harboring Tet-ON advanced vector and then transfected with pLVX-tight-puro vector encoding human LAMA1 with Lipofectamine 2000. After 1 day of transfection, culture medium was replaced with serum-free HuMEC containing 1 μg of doxycycline (Sigma) to collect secreted LAMA1 for 48 h. SUM159-LM1 cells (12,500 ml^–1^) were suspended in either CM (diluted two-to-five-fold in HuMEC) or HuMEC containing either 50 ng ml^–1^ activin B (R&D systems) or 1 μg ml^–1^ SCGB3A1 (R&D systems) and seeded in ultra-low attachment plates (Corning). Cells were cultured for 7 days, and oncospheres per well counted with a Zeiss Primovert microscope using the ×4 objective.

Gene responses to activin B and SCGB3A1 were analyzed in oncospheres. SUM159-LM1 cells were seeded in HuMEC/0.1% BSA on ultra-low attachment plates and treated with 50 ng ml^–1^ recombinant human activin B (R&D systems) for 6 h. To analyze responses to SCGB3A1, SUM159 cells were transduced with either pLVX-puro-hSCGB3A1 or control pLVX-puro lentiviral vectors and seeded on ultra-low attachment plates. Oncospheres were collected and gene expression analyzed by microarrays.

### Cell stimulation

Cancer cells were stimulated with CM from ECs or vascular niche factors to analyze the potential effect on TNC expression. MDA231 cancer cells were treated with ST1.6R-CM for 20 h and collected for analysis. For stimulation with recombinant activin B and OPG, MDA231 cells were serum starved using DMEM/0.5% FBS for 10 h and treated with either 50 ng ml^–1^ activin B (R&D Systems) or 200 ng ml^–1^ OPG (R&D Systems) for 20 h. For SCGB3A1 stimulation, HEK293T cells were transfected with either pLVX-puro-hSCGB3A1 or control pLVX-puro using Lipofectamine 2000. Transfected cells were cultured in DMEM/0.5% FBS for 36 h, and CM containing secreted SCGB3A1 was collected to treat serum-starved MDA231 cells for 20 h. For LN111 or LN121 stimulation, MDA231 cells were seeded on plates coated with LN111 or LN121 (10 μg ml^–1^, Biolamina) and cultured for 20 h. Total RNA from stimulated or unstimulated MDA231 cells was isolated and *TNC* expression analyzed by qPCR.

Macrophages were activated by TNC. Bone marrow-derived macrophages were serum starved (1% FBS and 20 μg ml^–1^ M-CSF) overnight and treated with either 3 μM TAK-242 (Merck Millipore) or vehicle control (0.1% DMSO in the final step) for 1 h before stimulation with recombinant TNC (2 μg ml^–1^, Merck Millipore) for 6 h. RNA was extracted, and gene expression assessed by qPCR or microarrays.

Lung ECs were stimulated with CM from different sources. To generate cancer cell-derived CM, MDA231-LM2 cells were cultured with DMEM/1% FBS for 24 h and the medium collected. To produce CM from neutrophils, undifferentiated HL60 cells were stimulated with 1.25% DMSO and 1 μM all-transretinoic acid (Sigma-Aldrich) to induce differentiation towards neutrophil lineage. Differentiated HL60 cells were washed with PBS and secreted proteins collected in RPMI 1640/1% FBS. To produce CM from macrophages, RAW264.7 cells were stimulated with 10 nM PMA for 24 h. Activated RAW264.7 cells were washed with PBS and secreted proteins collected in DMEM/1% FBS for EC stimulation. CM from unstimulated RAW264.7 cells was used as control.

Lung ECs were stimulated by TNC-activated macrophages via indirect coculture. ST1.6R cells were seeded on collagen I-coated plates and cultured with growth medium. Transwell polycarbonate membrane inserts (0.4-μm pore size, Corning) were coated with 10–20 μg ml^–1^ TNC (Merck) overnight at 4 °C. The culture medium of ST1.6R cells was replaced with M199 containing 1% FBS, and TNC-coated transwell inserts placed on ST1.6 culture plates. RAW264.7 cells were seeded onto TNC-coated transwell inserts. Indirect cocultures were incubated at 37 °C for 48–72 h before collection. Noncoated transwell inserts were used as controls.

Endothelial cells were stimulated with a collection of specific macrophage-derived factors. ST1.6R cells were serum starved (1% FBS) for 1 day and stimulated for 16–24 h with recombinant cytokines or other reagents as follows: 50 ng ml^–1^ CXCL1 (Peprotech), 50 ng ml^–1^ CXCL3 (Peprotech), 50 ng ml^–1^ CXCL10 (Peprotech), 100 ng ml^–1^ CCL2 (Peprotech), 100 ng ml^–1^ CCL3 (Biolegend), 100 ng ml^–1^ CCL5 (Peprotech), 100 ng ml^–1^ CCL12 (Biolegend), 100 ng ml^–10^ CCL22 (Biolegend), 10 ng ml^–1^ IL-1α (Peprotech), 10 ng ml^–1^ IL-1β (Peprotech), 100 ng ml^–1^ IL-6 (Peprotech), 100 ng ml^–1^ IL-12 (Peprotech), 100 ng ml^–1^ IL-15 (Peprotech), 100 ng ml^–1^ IL-18 (Peprotech), 100 ng ml^–1^ IL-23 (R&D systems), 100 ng ml^–1^ IL-27 (R&D systems), 100 ng ml^–1^ IL-35 (R&D systems), 10 ng ml^–1^ TNF-α (Peprotech), 100 ng ml^–1^ TNF-SF9 (Peprotech), 1 μg ml^–1^ CD40 (R&D systems) and 1 mM diethylenetriamine NONOate (abcam).

Recombinant TNC was used for direct stimulation of lung ECs. Human primary lung ECs were seeded on plates coated with 10 μg ml^–1^ recombinant TNC (Merck) in EBM-2 containing 0.2% FBS without supplements, and cultured for 16–20 h. Stimulated ECs were collected and expression of *INHBB*, *LAMA1*, *SCGB3A1* and *OPG* analyzed by qPCR.

### Lung EC adhesion on fibronectin and TNC substrates

Recombinant fibronectin (10 μg ml^–1^, R&D Systems) in PBS was loaded on glass coverslips precoated with poly-l-lysine (PLL; Sigma) and incubated overnight at 4 °C. After the PBS wash, coverslips were coated with 10 μg ml^–1^ recombinant TNC. ST1.6R cells were seeded onto the coverslips and incubated for 1 h at 37 °C, allowing cells to attach and spread. Images were taken using a Zeiss Primovert microscope equipped with Axiocam 105 color (Zeiss), and cell area measured by FIJI (ImageJ).

### Flow cytometry analysis

For analysis of general macrophages and neutrophils in mouse lung, the following antibodies were used: PE-conjugated anti-CD11b (1:3,000, BD Biosciences), Alexa647-conjugated anti-F4/80 (for macrophages; 1:400, eBioscience) or APC-conjugated anti-Gr1 (for neutrophils; 1:2,000, Invitrogen).

For staining of interstitial and alveolar macrophages we used the following: anti-CD45 conjugated with PE/Cy5 (1:3,000, eBioscience) or BV785 (1:300, Biolegend); anti-MerTK conjugated with PE (1:100, eBioscience) or PE/Cy7 (1:300, Biolegend); anti-CD64 conjugated with BV421 (1:100, Biolegend) or PE/Dazzle594 (1:300, Biolegend); anti-CD170 conjugated with Alexa647 (1:200, BD Pharmingen) or APC (1:300, Biolegend); and anti-CD11b conjugated with PE/Cy7 (1:1,000, eBioscience) or PE (1:300, Biolegend). Macrophage populations were as follows: alveolar, GFP^–^/CD45^+^/MerTK^+^/CD64^+^/CD170^+^/CD11b^–^; and interstitial, GFP^–^/CD45^+^/MerTK^+^/CD64^+^/CD170^–^/CD11b^+^. For analysis of immune responses in metastasis the following antibodies (all diluted 1:300 and from Biolegend) and reagents were used: T cell exhaustion, anti-CD3e-Alexa700, anti-PD1-BV421, anti-Lag3-PE/Cy7, anti-CD69-BV510, anti-Tim3-APC, anti-CTLA4-PE/Dazzle594 and anti-TIGIT-PE; Tregs, anti-CD45-BV785, anti-CD4-APC, anti-CD25-PerCP/Cy5.5 and anti-FoxP3-Alexa-488, with the FoxP3/Transcription Factor Fixation/Permeabilization Kit (eBioscience); NK cells, anti-CD45-PE and anti-CD49b-PE/Cy7; and neutrophils, anti-CD45-BV785, anti-CD11b-PE and anti-Ly6G-PerCP/Cy5.5. Macrophages in this setting were analyzed as described above.

Flow cytometry was performed on either LSR Fortessa with BD FACSDiva software 8.0.1 (BD Biosciences) or Attune NXT with Attune NXT analyzer software (Thermo Fisher Scientific). Data were analyzed using FlowJo v.10 software (Treestar).

### Gene expression profiles

Gene expression profiles were generated using either Affymetrix GeneChip Mouse Genome 430 v.2.0 or Human Genome U133 Plus v.2.0 Arrays, according to the manufacturer’s instructions. Raw CEL files were robust multiarray average (RMA) normalized and clustered with principle components using Chipster (v.3.8.0). Differential gene expression analysis was performed by two-group comparison using an empirical Bayes test with Benjamini–Hochberg correction of *P* values. Gene Ontology analysis was conducted with the Database for Annotation, Visualization and Integrated Discovery (DAVID)^60^, and the EC secretome gene set was generated utilizing GO:0005576 (GO term, extracellular region). To generate lung EC inflammation and proliferation signatures associated with metastasis (Supplementary Tables 3 and 4), genes of “HALLMARK_Inflammatory_Responce” and “REACTOME_Cell_Cycle_Mitotic” from the Molecular Signatures Database (MSigDB) of the Broad Institute were applied to gene expression profiles of metastasis-associated ECs, and genes with increased expression (*z*-score of “Week 3” minus “Control” >1) were selected to the signature. Heatmap images were produced using either MeV v.4.9.0 software or GraphPad Prism v.8, and volcano plots generated with R v.3.5. GSEA was performed with MSigDB. Nominal *P* values were calculated based on random gene set permutations with Benjamini–Hochberg correction. For violin plots, genes of specific gene sets described in each figure legend were *z*-scored and the average *z*-score for each gene from three biological replicates was calculated. Gene expression profiles of human metastasis (GSE14020 or lung metastasis samples from GSE14018)^61^ or primary tumor tissues (METABRIC discovery)^62^ from patients with breast cancer were normalized (RMA) using R v.3.5. For analysis of the correlation of stem cell gene sets with activin B signature (ActB-S, Supplementary Table 7) or SCGB3A1 signature (S3A1-S, Supplementary Table 8) in patients with breast cancer, we stratified samples from the METABRIC discovery dataset or patients with lung metastasis of GSE14018 based on ActB-S or S3A1-S expression (*z*-score). Patients with high or low expression of ActB-S or S3A1-S (upper and lower quantiles) were selected, and GSEA was conducted to analyze enrichment of stem cell gene sets in each group. Hierarchical clustering based on the expression of GSP58 was performed with the heatmap function in the R Stats Package using gene expression profiles of distant metastases of breast cancer (GSE14020 or lung metastasis samples from GSE14018). The clusters thus obtained were used for GSEA, lung metastasis-free survival analysis or correlation of *TNC* and signature of classically activated macrophages^36^. For analysis of the association of specific signatures with lung metastasis-free survival, we divided lung metastasis samples of GSE14018 into two groups—good or poor metastasis-free survival (based on upper and lower quantiles)—and used GSEA for analysis of the enrichment of GSP58, lung EC inflammation signatures associated with metastasis (Supplementary Table 4) or lung EC proliferation signature associated with metastasis (Supplementary Table 3). Pearson’s correlation analysis of vascular niche genes with *CDH5*, *TNC* or the signature of classically activated macrophages^36^ was conducted with GSE14020 using GraphPad Prism v.8. Gene expression data of a series of human breast cancer cell lines (Supplementary Table 9) were obtained from GSE16795 and RMA-normalized by R v.3.6. *TNC* expression and JNK signature^51^ were analyzed by *z*-score and compared with epithelial–mesenchymal status, as well as the breast cancer subtype of each cell line.

Survival analysis of patients with breast cancer was performed using datasets from METABRIC discovery, lung metastasis of GSE14018 or datasets compiled using the Kaplan–Meier plotter (KM plotter)^63^. The following datasets were used in the KM plotter: E-MTAB-365, GSE16716, GSE17907, GSE19615, GSE20271, GSE2034, GSE20711, GSE21653, GSE2603, GSE26971, GSE2990, GSE31519, GSE3494, GSE37946, GSE42568, GSE45255, GSE4611, GSE5327 and GSE7390 for relapse-free survival; and GSE16716, GSE20271, GSE20711, GSE3494, GSE37946, GSE42568, GSE45255 and GSE7390 for overall survival. The association of VEGF signature (ABE_VEGFA_TARGETS in the C2 collection)^42^ and TLR4 signature (GSE14769_UNSTIM_VS_240MIN_LPS_BMDM_UP in the C7 collection)^43^ with survival of patients with breast cancer was analyzed using the METABRIC discovery dataset.

### Immunohistochemistry and immunofluorescence

To study human metastases, 11 cases of breast cancer lung metastasis were identified using the database of the Institute of Pathology, University Hospital Heidelberg. Resected metastases from lungs were fixed in 10% buffered formalin for 24 h at room temperature and embedded in paraffin blocks. Tissue sections of 4-µm thickness were treated with CC2 buffer (pH 6.0) for antigen retrieval. Immunohistochemical analysis was performed using the following antibodies: anti-CD31 (ready-to-use, Roche), anti-SCGB3A1 (1:50, Bioss Antibodies) and anti-LAMA1 (1:50, Invitrogen). Automated immunostaining was done using Ventana Bench Mark Ultra automat with the OptiView DAB Kit (Roche), Dako AutostainerLink 48 and the EnVision Flex Kit (Agilent). Stained tissue sections were mounted with Consul-Mount (Thermo Fisher Scientific) and scanned by Aperio AT2 (Leica; magnification 1:400) for analysis. Quantification was performed using Aperio Image Scope v.11.2.0.780.

For analysis of metastatic nodules in mouse models, dissected lungs from mice harboring metastases were fixed in 10% buffered formalin for 4–12 h at 4 °C, incubated with 30% sucrose/PBS overnight at 4 °C and embedded in O.C.T. (Sakura Finetek). Sections (8 µm) were prepared with a Microm HM-525 cryotome (Thermo Fisher Scientific) and air-dried for 30 min at room temperature. Sections were washed with PBS, treated with blocking solution (0.5% Blocking Reagent (PerkinElmer), 0.1 M Tris-HCl (pH 7.5) and 0.15 M NaCl) and incubated with the following antibodies: anti-GFP (1:1,000, abcam), anti-CD31 (1:100, BD Pharmingen or 1:50, abcam), anti-cleaved caspase 3 (1:250 dilution, Cell Signaling), anti-Ki67 (1:200, Thermo Fisher Scientific), anti-F4/80 (1:100, Invitrogen), anti-TNC (1:4,000, Thermo Fisher Scientific), anti-VEGFR1 (1:25, R&D Systems) or anti-TNF-α (10 μg ml^–1^, R&D Systems). After washing, sections were stained with fluorescence-conjugated secondary antibodies and DAPI (BioLegend). Sections were mounted with Fluoromount-G (SouthernBiotech).

For vimentin immunostaining, cryosections from xenograft mouse lungs harboring metastases were rehydrated and quenched with 3% hydrogen peroxide. Antigen retrieval was carried out at 100 °C for 20 min with citrate buffer (pH 6.0, Vector Laboratories). Sections were treated with blocking solution, followed by incubation with anti-vimentin antibody (1:400, Leica Biosystems). Biotinylated anti-mouse IgG secondary antibody and the ABC avidin-biotin-DAB detection kit (Vector laboratories) were used for signal detection, according to the manufacturer’s instructions. Sections were counterstained with Mayer’s hematoxylin solution (Sigma-Aldrich) and mounted using Cytoseal XYL (Thermo Fisher Scientific). Images were obtained with a Cell observer microscope (Zeiss) equipped with either a Plan-Apochromat ×20/0.8 numerical aperture M27 or EC Plan-Neofluar objective lens (Zeiss) and analyzed with either FIJI (ImageJ) or ZEN imaging software (Zeiss).

### Immunocytochemistry

Either LN111 or LN121 (both 10 μg ml^–1^, Biolamina) was loaded on coverglass slips precoated with PLL (Sigma), and SUM159-LM1 cells were then seeded onto the coverslips. To inhibit integrin β1 function, anti-integrin β1 blocking antibody (2.5 μg ml^–1^, Merck) was used. After 24–48 h, cells were fixed with 2% formaldehyde/PBS on ice, permeabilized with 0.1% Triton X-100 and 0.1% Tween20, blocked and incubated with either anti-paxillin (1:1,000, BD Transduction Laboratories) or anti-cleaved caspase 3 (1:250, Cell signaling). Cy3- or Alexa-488-conjugated secondary antibodies (1:500, Invitrogen) were used to reveal staining and DAPI (BioLegend) to stain nuclei. Cells were imaged using a Cell observer microscope (Zeiss), and cell area and number of apoptotic cells per field were analyzed with FIJI (ImageJ).

### Ectopic expression and knockdown

To overexpress vascular niche genes, full-length complementary DNAs (cDNAs) of human INHBB and OPG were amplified by PCR from ST1.6R total cDNA. Human SCGB3A1 cDNA was synthesized as GeneArt Strings DNA Fragments (Invitrogen). cDNAs were subcloned into pLVX-Puro lentiviral expression vector (Clontech) and transfected into HEK293T cells with packaging plasmids psPAX2 and pMD2G, using Lipofectamine 2000. Viral supernatants were collected after 48 h and used to infect cancer cells in the presence of 8 μg ml^–1^ polybrene (Sigma-Aldrich). Infected cells were selected with 2 μg ml^–1^ puromycin (Invitrogen) for 7 days, and gene expression confirmed by qPCR. To generate cells overexpressing LAMA1, cancer cells were infected with lentivirus harboring pLVX-Tet-On Advanced vector (Clontech) and infected cells selected with Zeocin (Thermo Fisher Scientific) for 7 days, using 2 mg ml^–1^ for MDA231 and 500 μg ml^–1^ for SUM159. Human LAMA1 cDNA was purchased from Promega and subcloned into pLVX-Tight-puro vector (Clontech). Cancer cells with pLVX-Tet-On Advanced vector were transduced with pLVX-Tight-hLAMA1, and positive cells selected with 2 μg ml^–1^ puromycin. Zeocin- and puromycin-resistant cells were collected, and doxycycline-mediated inducible expression of LAMA1 was confirmed by qPCR.

The shERWOOD algorithm^64^ was utilized to design shRNAs to knock down human DR4, DR5 or INHBB; sequences can be found in Supplementary Table 11. Oligonucleotides were cloned into miR-E lentiviral vectors^65^. StdTomatoEP or StdTomatoEZ, modified versions of the original SGEP vector provided by J. Zuber in which the GFP reporter was replaced by the tdTomato protein and, as for StdTomatoEZ, the puromycin resistance cassette was replaced by the Zeocin resistance cassette. Constructs were transfected into HEK293T cells with psPAX2 and pMD2G using Lipofectamine 2000. The resulting lentiviruses were used to infect MDA231, MDA231-LM2 or ST1.6R cells. Knockdown efficiency was assessed by qPCR after selection by puromycin (2 μg ml^–1^) and/or Zeocin (600 μg ml^–1^). Human TNC was targeted as previously described^35^.

### ELISA and EIA

For detection of protein levels of INHBB, laminin α1 subunit, SCGB3A1 and OPG in lung ECs, mouse lungs harboring MDA231-LM2 metastasis were harvested and ECs purified by FACS. Lung ECs were suspended in PBS supplemented with 1× HALT protease and phosphatase inhibitor cocktail (Thermo Fisher Scientific) and lysed by sonication with SONIFIER W-250 D (BRANSON). Homogenates were pelleted, and supernatants used for protein analysis. Kits for mouse inhibin-B EIA (Raybiotech), mouse LAMA1 ELISA (FineTest), mouse HIN-1/SCGB3A1 ELISA (Raybiotech) and mouse OPG ELISA (Raybiotech) were used according to the manufacturers’ instructions. Total protein concentration of each sample was determined by BCA protein assay kit (Pierce), and the amount of each factor in the samples was calculated using standard curves.

### qPCR

Total RNA was extracted using either the RNeasy Mini kit (Qiagen) or Arcturus PicoPure RNA isolation kit (Applied Biosystems), and reverse transcription was performed with the High-Capacity cDNA Reverse Transcription kit (Applied Biosystems) according to the manufacturers’ instructions. qPCR was performed with the SYBR Green gene expression assay (Applied Biosystems) using the ViiA 7 Real-Time PCR System (Applied Biosystems) and analyzed with Quant Studio Real-Time PCR software v.1.3 (Applied Biosystems). Primer pairs are listed in Supplementary Table 11.

### Immunoblot analysis

MDA231-LM2 cells, treated with incremental dosages of recombinant OPG (10–200 ng ml^–1^, R&D Systems) in the presence of 50 ng ml^–1^ recombinant TRAIL (R&D Systems), were lysed in RIPA buffer with 1× HALT protease and phosphatase inhibitor cocktail (Thermo Fisher Scientific). Immunoblots were performed as previously reported^51^. Primary antibodies used were as follows: anti-cleaved caspase 3 (1:500), anti-caspase 3 (1:1,000) and anti-vinculin (1:1,000), all from Cell Signaling. Incubation with horseradish peroxidase-conjugated IgG (1:10,000, Leica), followed by Clarity Western ECL Substrate (Bio-Rad) and exposure to X-ray films (Fuji-film) was used to develop the signal.

### Statistics and reproducibility

Statistical analyses were performed as described in individual figure legends. Generally, *P* < 0.05 was considered significant and statistical tests for in vitro experiments were two-tailed, unless otherwise indicated. All functional in vivo experiments were based on substantial previous in vitro results that indicated one-directional effect, and thus one-tailed tests were adopted for these experiments. Statistics for average *z*-scores of gene signatures were conducted with one-way analysis of variance (ANOVA) and Dunnett’s multiple comparison test, unless otherwise indicated. When applicable, data distribution was assumed to be normal but this was not formally tested. For Kaplan–Meier analyses in patients with breast cancer, statistical differences were calculated by log-rank (Mantel–Cox) test. The GSE14020 gene expression dataset was used to study the correlation between vascular niche genes and *Cdh5*, *Tnc* or signature of activated macrophages in a cohort of 65 metastasis samples from patients with breast cancer. Gene expression values for each gene within individuals were associated in a correlation matrix. Next, Pearson correlation coefficient (*r*) and *P* values were calculated for each comparison. Statistical significance of gene expression data from microarrays was calculated with Chipster. Statistical analyses of GO and gene set enrichment were conducted using DAVID^60^ and GSEA, respectively. For GSEA, false discovery rate (FDR) < 0.25 was considered statistically significant. All other statistical analyses were performed using GraphPad Prism v.8 for Windows. Exact *P* values not indicated in figures are summarized in Supplementary Table 12.

Biological replicates for each experiment are noted in figure legends. Although statistical methods were not used to predetermine sample sizes, this was generally determined based on previous studies involving similar experiments^35,51,66^. No data were excluded from the analyses. Mice were randomized from different cages and allocated to control and treatment groups for metastasis experiments. Immunohistochemistry and immunofluorescence images were acquired and analyzed in a blinded fashion. For other experiments, neither randomization nor blinding was used.

### Reporting Summary

Further information on research design is available in the Nature Research Reporting Summary linked to this article.

## Supplementary information

Reporting Summary

Supplementary Tables 1–12. Multiple-tab Excel workbook.

## Data Availability

All transcriptomic datasets generated in this study have been deposited at the NCBI Gene Expression Omnibus under accession code GSE156354. Source data are provided with this paper. All other data supporting the findings of this study are available within the article and its Supplementary Information or from the corresponding author on reasonable request.

## Source data

Source Data Fig. 1 Statistical Source data.
Source Data Fig. 2 Statistical Source data.
Source Data Fig. 3 Statistical Source data.
Source Data Fig. 4 Statistical Source data.
Source Data Fig. 5 Statistical Source data.
Source Data Fig. 5 Unprocessed western blots.
Source Data Fig. 6 Statistical Source data.
Source Data Fig. 7 Statistical Source data.
Source Data Fig. 8 Statistical Source data.
Source Data Extended Data Fig. 1 Statistical Source data.
Source Data Extended Data Fig. 2 Statistical Source data.
Source Data Extended Data Fig. 3 Statistical Source data.
Source Data Extended Data Fig. 4 Statistical Source data.
Source Data Extended Data Fig. 5 Statistical Source data.
Source Data Extended Data Fig. 6 Statistical Source data.
Source Data Extended Data Fig. 7 Statistical Source data.
Source Data Extended Data Fig. 8 Statistical Source data.
Source Data Extended Data Fig. 9 Statistical Source data.
Source Data Extended Data Fig. 10 Statistical Source data.
