# Supplementary material for: Perivascular tenascin C triggers sequential activation of macrophages and endothelial cells to generate a pro-metastatic vascular niche in the lungs
Source: Nat Cancer. 2022 Apr 25;3(4):486–504. doi: 10.1038/s43018-022-00353-6 (PMC9046090; doi:10.1038/s43018-022-00353-6)
Supplement: Supplementary file 1 — Reporting Summary [file 43018_2022_353_MOESM1_ESM.pdf]

nature research

Corresponding author(s): Thordur Oskarsson

Last updated by author(s): Feb 22, 2022

Reporting Summary

Nature Research wishes to improve the reproducibility of the work that we publish. This form provides structure for consistency and transparency in reporting. For further information on Nature Research policies, see our Editorial Policies and the Editorial Policy Checklist.

Statistics

For all statistical analyses, confirm that the following items are present in the figure legend, table legend, main text, or Methods section.

n/a	Confirmed	
☐	☒	The exact sample size (n) for each experimental group/condition, given as a discrete number and unit of measurement
☐	☒	A statement on whether measurements were taken from distinct samples or whether the same sample was measured repeatedly
☐	☒	The statistical test(s) used AND whether they are one- or two-sided<br>*Only common tests should be described solely by name; describe more complex techniques in the Methods section.*
☒	☐	A description of all covariates tested
☐	☒	A description of any assumptions or corrections, such as tests of normality and adjustment for multiple comparisons
☐	☒	A full description of the statistical parameters including central tendency (e.g. means) or other basic estimates (e.g. regression coefficient) AND variation (e.g. standard deviation) or associated estimates of uncertainty (e.g. confidence intervals)
☐	☒	For null hypothesis testing, the test statistic (e.g. F, t, r) with confidence intervals, effect sizes, degrees of freedom and P value noted<br>*Give P values as exact values whenever suitable.*
☒	☐	For Bayesian analysis, information on the choice of priors and Markov chain Monte Carlo settings
☒	☐	For hierarchical and complex designs, identification of the appropriate level for tests and full reporting of outcomes
☐	☒	Estimates of effect sizes (e.g. Cohen's d, Pearson's r), indicating how they were calculated

Our web collection on statistics for biologists contains articles on many of the points above.

Software and code

Policy information about availability of computer code

Data collection

- Living Image (version 4.4)
- Quant Studio Real-Time PCR Software v1.3
- BD FACSDiva Software (version 8.0.1)

Data analysis

- GraphPad Prism 8 (version 8.4.1)
- Chipster (version 3.8)
- GSEA Software (version 4.0.3)
- Database for Annotation, Visualization and Integrated Discovery (DAVID) (version 6.8)
- R (version 3.5 & 3.6)
- ZEN (black edition 2.1)
- Fiji extension of the ImageJ software (version 2.0.0)
- Living Image (version 4.4)
- Quant Studio Real-Time PCR Software v1.3
- FlowJo (version 10.6.1)
- MeV (version 4.9.0)

For manuscripts utilizing custom algorithms or software that are central to the research but not yet described in published literature, software must be made available to editors and reviewers. We strongly encourage code deposition in a community repository (e.g. GitHub). See the Nature Research guidelines for submitting code & software for further information.

Data

Policy information about availability of data

All manuscripts must include a data availability statement. This statement should provide the following information, where applicable:

- Accession codes, unique identifiers, or web links for publicly available datasets
- A list of figures that have associated raw data
- A description of any restrictions on data availability

The datasets generated and/or analyzed for the current study are available in the GEO repository (https://www.ncbi.nlm.nih.gov/geo/browse/) under the Accession names: GSE156354 (datasets generated in this study), GSE14020, GSE14018, GSE14769, GSE16795 (datasets analyzed in this study). Compiled datasets used in KM plotter: E-MTAB-365, GSE16716, GSE17907, GSE19615, GSE20271, GSE2034, GSE20711, GSE21653, GSE2603, GSE26971, GSE2990, GSE31519, GSE3494, GSE37946, GSE42568, GSE45255, GSE4611, GSE5327 and GSE7390 for relapse-free survival; GSE16716, GSE20271, GSE20711, GSE3494, GSE37946, GSE42568, GSE45255 and GSE7390 for overall survival. METABRIC discovery dataset (Curtis C. et al., Nature, 2012) was used for correlation analysis of breast cancer patient survival with VEGF/TLR4 signature, and for the stem cell signatures with ActB-S or S3A1-S. Gene set signatures analyzed in the current study are available in Molecular Signatures Database of the Broad Institute (https://www.gsea-msigdb.org/gsea/msigdb/).

Field-specific reporting

Please select the one below that is the best fit for your research. If you are not sure, read the appropriate sections before making your selection.

☒ Life sciences ☐ Behavioural & social sciences ☐ Ecological, evolutionary & environmental sciences

For a reference copy of the document with all sections, see nature.com/documents/nr-reporting-summary-flat.pdf

Life sciences study design

All studies must disclose on these points even when the disclosure is negative.

Sample size	Statistical methods were not used to pre-determine sample sizes, however this was generally determined based on previous studies involving similar experiments (Oskarsson T. et al., Nat Med, 2011; Insua-Rodriguez J. et al., Embo Mol Med, 2018; Pein M. et al., Nat Commun, 2020).
Data exclusions	No data were excluded from the analyses.
Replication	Biological replicates for each experiment are noted in figure legends.
Randomization	Mice were randomized from different cages and allocated to control and treatment groups for metastasis experiments. Other experiments were not randomized.
Blinding	Immunohistochemistry and immunofluorescence images were acquired and analyzed in a blinded fashion. For other experiments blinding was not used.

Reporting for specific materials, systems and methods

We require information from authors about some types of materials, experimental systems and methods used in many studies. Here, indicate whether each material, system or method listed is relevant to your study. If you are not sure if a list item applies to your research, read the appropriate section before selecting a response.

Materials & experimental systems

n/a	Involved in the study	
☐	☒	Antibodies
☐	☒	Eukaryotic cell lines
☒	☐	Palaeontology and archaeology
☐	☒	Animals and other organisms
☐	☒	Human research participants
☒	☐	Clinical data
☒	☐	Dual use research of concern

Methods

n/a	Involved in the study	
☒	☐	ChIP-seq
☐	☒	Flow cytometry
☒	☐	MRI-based neuroimaging

Antibodies

Antibodies used	Flow cytometry:<br>- Anti-mouse CD45-PE, 1:3000, eBiosciences, Cat. 12-0451-83, Clone 30-F11<br>- Anti-mouse CD45-PE, 1:300, Biolegend, Cat. 103106, Clone 30-F11<br>- Anti-mouse CD45-BV785, 1:300, Biolegend, Cat. 103149, Clone 30-F11

- Anti-mouse CD3e-AF700, 1:300, Biolegend, Cat. 152315, Clone 500A2
- Anti-mouse CD4-APC, 1:300, Biolegend, Cat. 100411, Clone GK1.5
- Anti-mouse CD11b-PE, 1:300, Biolegend, Cat. 101207, Clone M1/70
- Anti-mouse CD11b-PE, 1:3000, BD Biosciences, Cat. 553311, Clone M1/70
- Anti-mouse CD11b-PE/Cy7, 1:1000, eBioscience, Cat. 25-0112-82, Clone M1/70
- Anti-mouse CD25-PerCP/Cy5.5, 1:300, Biolegend, Cat. 101911, Clone 3C7
- Anti-mouse CD49b-PE/Cy7, 1:300, Biolegend, Cat. 108921, Clone DX5
- Anti-mouse CD64-BV421, 1:100, Biolegend, Cat. 139309, Clone X54-5/7.1
- Anti-mouse CD64-PE/Dazzle594, 1:300, Biolegend, Cat. 139319, Clone X54-5/7.1
- Anti-mouse CD69-BV510, 1:300, Biolegend, Cat. 104531, Clone H1.2F3
- Anti-mouse CD152 (CTLA4)-PE/Dazzle594, 1:300, Biolegend, Cat. 106317, Clone UC10-4B9
- Anti-mouse F4/80-Alexa647, 1:400, eBioscience, Cat. 51-4801-80, Clone BM8
- Anti-mouse FoxP3-AF488, 1:300, Biolegend, Cat. 126406, Clone MF-14
- Anti-mouse CD223 (Lag3)-PE/Cy7, 1:300, Biolegend, Cat. 125226, Clone C9B7W
- Anti-mouse Ly6G-PerCP/Cy5.5, 1:300, Biolegend, Cat. 127615, Clone 1A8
- Anti-mouse MerTK-PE/Cy7, 1:300, Biolegend, Cat. 151521, Clone 2B10C42
- Anti-mouse MerTK-PE, 1:100, eBioscience, Cat. 12-5751-82, Clone DS5MMER
- Anti-mouse CD279 (PD1)-BV421, 1:300, Biolegend, Cat. 135217, Clone 29F.1A12
- Anti-mouse CD170-Alexa647, 1:200, BD Pharmingen, Cat. 562680, Clone E50-2440
- Anti-mouse CD170-APC, 1:300, Biolegend, Cat. 155507, Clone S17007L
- Anti-mouse TIGIT-PE, 1:300, Biolegend, Cat. 142103, Clone 1G9
- Anti-mouse CD366 (Tim3)-APC, 1:300, Biolegend, Cat. 134007, Clone B8.2C12
- Anti-mouse CD326 (EpCAM)-PE, 1:250, eBioscience, Cat. 12-5791-83, Clone G8.8
- Anti-mouse CD140a-APC, 1:50, eBioscience, Cat. 17-1401-81, Clone APA5
- Anti-mouse CD140b-APC, 1:50, eBioscience, Cat. 17-1402-82, Clone APB5
- Anti-mouse CD31-PE-Cy7, 1:500, eBioscience, Cat. 25-0311-82, Clone 390
- Anti-mouse Gr1-APC, 1:2000, Invitrogen, Cat. 17-5931-82, Clone RB6-8C5

Magnetic-activated cell sorting:
- Anti-mouse CD31, 1:25, BD Pharmingen, Cat. 550274, Clone Mec13.3

Immunohistochemistry:
- Anti-human Vimentin, 1:400, Novocastra™, Leica Biosystemsclone, product code NCL-L-VIM-572, Clone SRL-33
- Anti-human CD31, Ready-to-use, Roche, Cat. 05463475001
- Anti-human SCGB3A1, 1:50, Bioss Antibodies, Cat. bs-6397R
- Anti-human LAMA1, 1:50, Invitrogen, Cat. PA5-111038

Immunofluorescence:
- Anti-GFP, 1:1000, abcam, Cat. ab290
- Anti-GFP, 1:1000, abcam, Cat. ab13970
- Anti-mouse CD31, 1:100, BD Pharmingen, Cat. 550274, Clone Mec13.3
- Anti-mouse CD31, 1:50, abcam, Cat. ab28364
- Anti-mouse Cleaved caspase 3, 1:250, Cell Signaling, Cat. 9579, Clone D3E9
- Anti-mouse F4/80, 1:100, Invitrogen, Cat. 14-4801-82, Clone BM8
- Anti-mouse TNF, 10 ug/ml, R&D Systems, Cat. AF410
- Anti-human TNC, 1:4000, ThermoFisher Scientific, Cat. MA1-26779, Clone BC-24
- Anti-human Ki67, 1:200, ThermoFisher Scientific, Cat. 14-5698-82, Clone SolA15
- Anti-mouse VEGFR1, 1:25, R&D Systems, Cat. AF471

Immunocytochemistory
- Anti-Paxillin, 1:1000, BD Transcuction Laboratories, Cat. 610051
- Anti-Cleaved caspase 3, 1:250, Cell Signaling, Cat. 9579, Clone D3E9

Western blot:
- Anti-human Cleaved caspase 3, 1:500, Cell Signaling, Cat. 9664, Clone 5A1E
- Anti-human Caspase 3, 1:1000, Cell Signaling, Cat. 9662
- Anti-human Vinculin, 1:1000, Cell Signaling, Cat. 4650

Neutralizing Antibodies:
- Anti-human/mouse VEGFA, 5 or 10 mg/kg, provided from Genentech, clone B20.4.1.1
- Anti-mouse VEGFR2, 40 mg/kg, BioXcell, Cat. BE0060, Clone DC101
- Anti-mouse Ly6G, 20 mg/kg, BioXcell, Cat. BE0075-1, Clone 1A8
- Anti-human Integrin beta1, 2.5 ug/ml, Merck, Cat. MABT409, Clone AIIB2

Validation

Primary antibodies used in this study have been validated by the respective companies for the respective species and applications. We used isotype controls as negative controls. Validation statement for each primary antibody is provided on the manufacture's website. Anti-human/mouse VEGFA antibody (B20.4.1.1) was provided by Genentech and validated in previous publication (Liang W.C. et al., J Biol Chem, 2006).

Eukaryotic cell lines

Policy information about cell lines

Cell line source(s)

MDA-MB-231, 4T1, HEK293T, RAW264.7 and HL60 cells were from ATCC. MDA231-LM2 was generated by the Massagué lab by in vivo selection using the breast cancer cell line MDA-MB-231 (Minn et al., Nature, 2005). E0771 cells were obtained from

	CH3 BioSystems. SUM159 cell line was obtained from Asterand Bioscience. SUM159-LM1 was generated by the Oskarsson lab by in vivo selection using the breast cancer cell line SUM159 (Insua-Rodríguez et al, EMBO Mol Med, 2018). ST1.6R cell line was generated and provided by R. E. Unger and C. J. Kirkpatrick (Unger et al., Microvascular Res, 2002).
Authentication	Cell lines were authenticated using Multiplex Cell Authentication (MCA) by Multiplexion (Heidelberg, Germany). Cell line Identification was done using Single Nucleotide Polymorphism (SNP)-profiling and the complete genotype information is compared to a reference database of currently 850 distinct reference cell lines authenticated Short tandem repeat (STR) Profiling.
Mycoplasma contamination	The purity of cell lines was validated using the Multiplex cell Contamination Test (McCT) by Multiplexion (Heidelberg, Germany). Contaminations are detected with specific primer sequences in a multiplex PCR targeting cellular, bacterial and viral genome regions followed by hybridization using specific oligonucleotide probes. No Mycoplasma, SMRV or interspecies contamination was detected.
Commonly misidentified lines (See ICLAC register)	No cell lines listed in the ICLAC database were used.

Animals and other organisms

Policy information about studies involving animals; ARRIVE guidelines recommended for reporting animal research

Laboratory animals	NOD Scid gamma (NSG) mice from in-house breeding, BALB/c mice (Janvier Labs or Envigo), and C57BL/6 mice (Jackson Laboratory) were used for experiments. Only female mice were used for the study and mice were 6-12 weeks of age. Mice were housed in individually ventilated cages with control of temperature (approx. 22 ºC) and humidity (50%) under 12-12 h light-dark cycle.
Wild animals	The study did not involve wild animals.
Field-collected samples	The study did not involve samples collected from the field.
Ethics oversight	Animal care and procedures were approved by the governmental review board of the state of Baden-Wuerttemberg, Regierungspraesidium Karlsruhe, and followed the German legal regulations.

Note that full information on the approval of the study protocol must also be provided in the manuscript.

Human research participants

Policy information about studies involving human research participants

Population characteristics	The study involves human material of archived lung metastasis tissue sections. Archived samples were acquired from the National Center for Tumor Diseases (NCT) in Heidelberg. The samples correspond to tissue sections of lung metastasis lesions from female breast cancer patients.
Recruitment	11 cases of breast cancer metastasis in lungs were identified using the database of the Institute of Pathology in University Hospital Heidelberg. Patients gave written informed consent.
Ethics oversight	The study on human breast cancer metastases were approved by the ethics committee of the University of Heidelberg Medical Faculty (S-716/2018) and conformed to the principles of the WMA Declaration of Helsinki and the Department of Health and Human Service Belmont Report.

Note that full information on the approval of the study protocol must also be provided in the manuscript.

Flow Cytometry

Plots

Confirm that:

☒ The axis labels state the marker and fluorochrome used (e.g. CD4-FITC).

☒ The axis scales are clearly visible. Include numbers along axes only for bottom left plot of group (a 'group' is an analysis of identical markers).

☒ All plots are contour plots with outliers or pseudocolor plots.

☒ A numerical value for number of cells or percentage (with statistics) is provided.

Methodology

Sample preparation	For flow cytometric analysis and sorting of cells obtained from mouse lungs, lungs were digested at 37 °C in PBS supplemented with 0.5 % Collagenase, 1 % Dispase and 30 ug/ml DNase for 45 min. Single cell suspensions were obtained by filtering through 70 um nylon filters and red blood cells were lysed with ACK lysis buffer. Cells were then counted on a ViCell Automated Cell Counter to ensure use of sufficient amounts of antibodies. Mouse FcR Blocking Reagent was used to block unwanted binding of antibodies to mouse cells expressing Fc receptors for 10 min on ice. Cells were then stained for 30 min on ice with the fluorescence-conjugated antibodies in the dark. Afterwards, cells were washed three times, resuspended in FACS buffer contaning DAPI.

Instrument	FSR Fortessa (BD), FACSAria I (BD), FACSAria II (BD) or Attune NXT (ThermoFisher Scientific)
Software	Data collection:<br>BD FACSDiva Software version 8.0.1 (BD) or Attune NXT analyzer (ThermoFisher Scientific)<br>Data analysis:<br>FlowJo version 10.6.1
Cell population abundance	Sorting purities were evaluated by acquisition of post-sort fractions directly after the sort using the same gates for sorting. Purities were generally above 90 %.
Gating strategy	Gating strategy for lung endothelial cell isolation is shown in Extended Data Fig. 1c. Briefly, cells were gated on FSC-A/SSC-A, doublets were excluded by FSC-H/FSC-A and dead cells were excluded by DAP stain. Gating for DAPI was determined using unstained control. Cancer cell exclusion was done by gating on GFP-negative cell fraction, and the gate boundary was determined by acquisition of a healthy lung sample that does not contain GFP-positive cancer cells. Endothelial cells were gated on the PE-negative, APC-negative and PE-Cy7-positive fraction, where PE and APC stain were panels of negative selection markers of which individual antibody concentrations had been adjusted to similar fluorescence intensities during acquisition. Gates for PE-Cy7-positive fractions were set based on PE-Cy7-unstained controls and confirmed with PE-Cy7-conjugated isotype controls to ensure specific staining.<br>For gating strategies of lung macrophages and neutrophils, cells were gated on FSC-A/SSC-A, doublets were excluded by FSC-H/FSC-A, dead cells were excluded by DAPI stain, and F4/80-Alexa647-positive macrophages, MerTK/CD64-positive macrophages, MerTK/CD64/CD170-positive and CD11b-negative alveolar macrophages, MerTK/CD64/CD11b-positive and CD170-negative interstitial macrophages or Ly6G-APC-positive/CD11b-PE-positive neutrophils were gated.<br>For gating strategies of T-cells with exhaustion markers, cells were gated on FSC-A/SSC-A, doublets were excluded by FSC-H/FSC-A, dead cells were excluded by Live-dead staining, and CD3e-positive T-cells with exhaustion markers (Lag3, CD69, CTLA4, PD1, TIGIT or Tim3) were gated.<br>For gating strategy of regulatory T-cells and NK cells, cells were gated on FSC-A/SSC-A, doublets were excluded by FSC-H/FSC-A, dead cells were excluded by Live-dead staining, and CD4/CD25/FOXP3-positive cells (regulatory T-cells) or CD45/CD49b-positive cells (NK cells) were gated.

☒ Tick this box to confirm that a figure exemplifying the gating strategy is provided in the Supplementary Information.
