# Supplementary material for: Perivascular tenascin C triggers sequential activation of macrophages and endothelial cells to generate a pro-metastatic vascular niche in the lungs
Source: Nat Cancer. 2022 Apr 25;3(4):486–504. doi: 10.1038/s43018-022-00353-6 (PMC9046090; doi:10.1038/s43018-022-00353-6)

Source data

Related to Fig. 5b
Uncropped western blot images for caspase 3, cleaved caspase 3 and vinculin.
Red boxes indicate the area used for figure.

Caspase 3 and Cleaved caspase 3

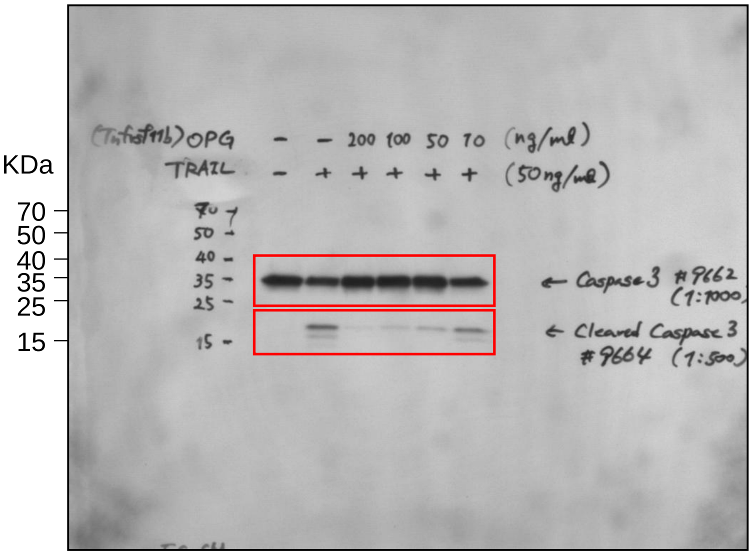

Vinculin

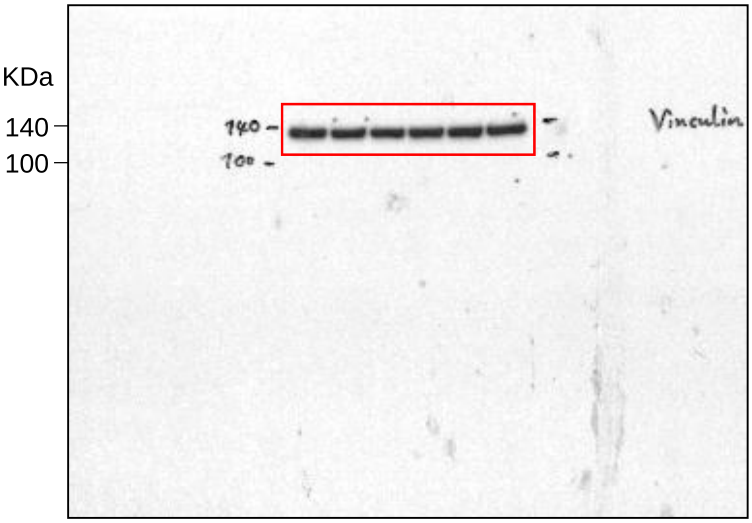

Supplement: Source Data Fig. 5 — Unprocessed western blots. [file 43018_2022_353_MOESM8_ESM.pdf]
